# Density-Potential Functional Theory of Electrochemical Double Layers: Calibration on the Ag(111)-KPF_6_ System and Parametric Analysis

**DOI:** 10.1021/acs.jctc.2c00799

**Published:** 2023-01-18

**Authors:** Jun Huang

**Affiliations:** Institute of Energy and Climate Research, IEK-13: Theory and Computation of Energy Materials, Forschungszentrum Jülich GmbH, 52425Jülich, Germany

## Abstract

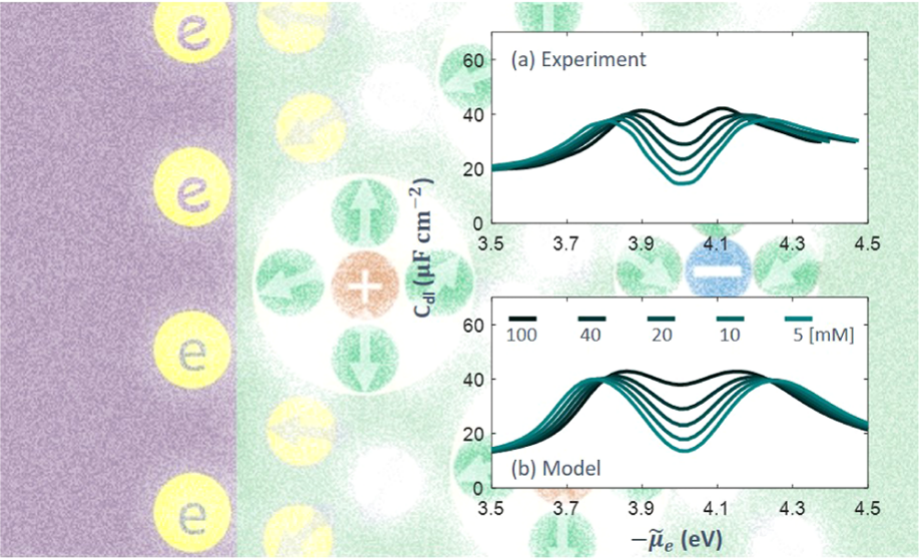

The density-potential functional theory (DPFT) of electrochemical double layer (EDL) is upgraded by adopting (generalized) gradient approximations for kinetic, exchange, and correlation functionals of metal electrons. A new numerical scheme that is more stable and converges faster is proposed to solve the DPFT model. The DPFT model is calibrated with existing differential double-layer capacitance (*C*_dl_) data of the EDL at Ag(111)-KPF_6_ aqueous interface at five concentrations at room temperature. Metal electronic effects are essential to explain why the two peaks of the camel-shaped *C*_dl_ curves are almost symmetric in spite of the size difference of the hydrated cations and anions. A systematic parametric analysis is then conducted in terms of key EDL properties, including the potential of zero charge and the differential capacitance. The parametric analysis, on the one hand, elucidates how quantum mechanical behaviors of metal electrons as well as interactions between metal electrons and the electrolyte solution impact the EDL properties and, on the other hand, identifies key parameters of the DPFT model, which should be calibrated using first-principles calculations and/or advanced experiments in the future.

## Introduction

Over the past few years, we have been developing a density-potential functional theory (DPFT) for electrochemical double layers (EDLs), so named because the grand potential of EDLs is a hybrid functional in terms of the electron density and the electric potential.^1−3^ Broadly speaking, DPFT belongs to semiclassical theories of EDLs, along with joint density functional theory (JDFT)^4−9^ and DFT-reference interaction site model (DFT-RISM).^10−13^ The key feature distinguishing DPFT from other semiclassical models is that the kinetic energy of electrons is expressed as an explicit functional of electron density rather than calculated from wave functions of an auxiliary noninteracting electronic system in the Kohn–Sham DFT. Such an idea can be traced back to the Thomas–Fermi theory of an electron gas in the 1920s, which is widely termed orbital-free DFT (OFDFT) nowadays.^14,15^

OFDFT has many developments and applications, notably including the proof of stability of matter using the Thomas–Fermi–von Weizsäcker (TFvW) theory of electronic gas,^16^ theory of work function and surface energy of metal surfaces,^17−19^ computational modeling of materials,^14,15,20^ and hydrodynamic theory of quantum plasmonics.^21−24^ Inspired by earlier works on metal surfaces,^17−19^ the jellium model of EDL developed in the 1980s also employed the OFDFT to describe metal electronic effects.^25−29^

As the kinetic energy functional can be, at best, approximated, OFDFT can by no means surpass Kohn–Sham DFT in terms of accuracy in computing material properties, though encouraging progress of improving the OFDFT accuracy has been reported.^30−33^ That said, OFDFT has unique merits that make it attractive to the problem of modeling EDLs. First and foremost, combined with a classical DFT of the electrolyte solution, OFDFT allows a grand canonical, namely, constant-potential description of EDLs. Second, the low computational cost of OFDFT allows us to model realistic EDLs at nanoparticles that will be beyond the reach of Kohn–Sham DFT in years to come. In short, OFDFT offers a viable approach to tackle two grand challenges of modeling EDLs, namely, treatment of the electrode potential and simulation of EDLs of realistic scales. EDL, in turn, provides an exciting playground for OFDFT to realize its full potential.

In addition to the electronic structure, equally important to EDL models is the electrolyte solution, which can be treated on varying levels of complexity, including the Debye–Hückel theory, the Poisson–Boltzmann theory, and its variants such as the modified Poisson–Boltzmann theory, and integral equation theories such as the reference interaction site model, see recent review articles.^34−36^ In DPFT, the free energy functional of the electrolyte solution was derived rigorously from statistical field theory and is exactly on the mean-field level.^2^ Compared to the Poisson–Boltzmann theory that is most frequently used in joint DFTs, the obtained free energy functional further considers ion size effects, orientational polarization of solvent molecules, and other short-range interactions using a reference system.

DPFT is different from earlier jellium models of EDL in several aspects. First, previous jellium models were developed and solved under constant-charge conditions, namely, conditions where the number of metal electrons is preset.^25−29^ Therefore, the double-layer capacitance calculated from these jellium models was often plotted as a function of the electrode charge. On the contrary, DPFT is a constant-potential model which is able to simulate EDLs as a function of electrode potential. Second, previous jellium models inherited Smith’s treatment of the metal surface.^17^ Specifically, the metal electrons were described using the Thomas–Fermi–Dirac–Wagner theory without gradient terms, and the electron density was solved approximately using trail functions. In comparison, DPFT upgrades the description of metal electrons by including gradient terms, and solves for the electron density and the conjugate electric potential self-consistently using a numerical scheme. Note in passing that the trail functions are limited to planar, one-dimensional (1D) EDLs, while the numerical scheme is, in principle, applicable to EDLs of arbitrary structures. Third, as for the electrolyte solution, previous jellium models used either the Poisson–Boltzmann theory or the integral equation theory of liquid, while DPFT introduces a general statistical field theory framework into which many effects and interactions that were neglected before can be incorporated.

This paper is the fourth one in the series. The first paper introduced the first version of DPFT that used the Thomas–Fermi–Weizsäcker theory for the kinetic energy functional, the Dirac–Wigner (DW) theory for the exchange–correlation energy functional, and a modified Poisson–Boltzmann theory for the electrolyte solution.^1^ Constant-potential simulations of metal–solution interfaces were conducted, from which the potential of zero charge (pzc) was obtained and analyzed in terms of electrolyte effects. In the second paper, we introduced the discrete structure of metal cationic cores, considered specific adsorption of electrolyte ions using a model Hamiltonian approach, and compared the model with experimental data measured on Ag(111)-aqueous KPF_6_ solution interfaces.^3^ In the third paper, we focused on improving the treatment of the electrolyte solution and derived a comprehensive mean-field free energy functional by following the statistical field theory procedure.^2^

The purpose of this paper is 4-fold. First, we modify the theory by upgrading the exchange–correlation energy functional from the local density approximation level to the generalized gradient approximation (GGA) level and using Morse potentials to describe metal–solution interactions. Second, we improve the numerical stability and efficiency of solving DPFT by proposing a new numerical scheme. Specifically, we solve for the cubic root of the electron density rather than the electron density itself, which avoids numerical problems encountered in calculating terms of the electron density with fractional exponents. In addition, we implement the model in COMSOL Multiphysics, rather than Matlab previously, because it is more convenient to model EDLs of various structures in COMSOL Multiphysics. Third, we calibrate the model with high-quality experimental data of the EDL at the Ag(111)-KPF_6_ aqueous interface at five concentrations.^37^ Comparison between this model and classical EDL models reveals the essential roles of metal electronic effects in understanding detailed features of the experimental data. Fourth, using the calibrated model as the baseline, we conduct a systematic parametric analysis of the model, guiding where further improvements of DPFT should be targeted.

## Modifications to DPFT

**Figure 1:**
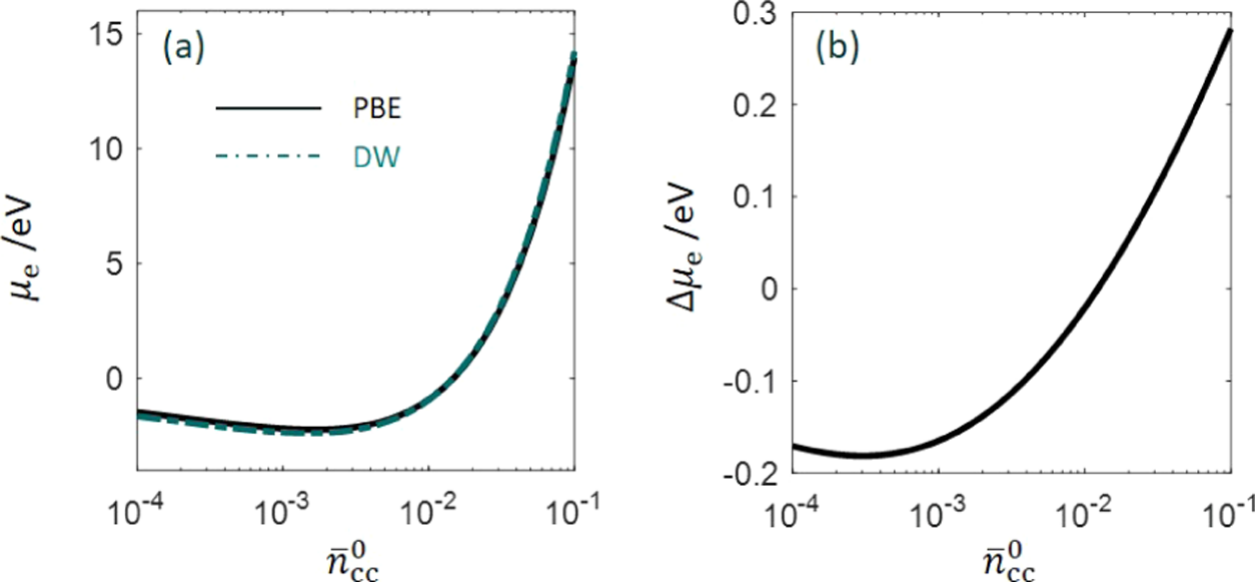
(a) Chemical potential of electrons in metal bulk calculated using the PBE functional and the Dirac–Wigner (DW) functional for the exchange–correlation energy. The kinetic energy is described using the Thomas–Fermi–von Weizsäcker (TFvW) functional in both cases. The difference between the two functionals is shown in panel (b). *n̅*_cc_^0^ is the dimensionless metal cationic charge density normalized to a reference number density (*a*_0_)^−3^, with *a*_0_ being the Bohr radius.

We exposit the DPFT framework in the Supporting Information of this paper and mention modifications made in this paper herein. In the quantum part, the exchange–correlation functional is upgraded from the Dirac–Wigner (DW) functional, a local density approximation, to the Perdew–Burke–Ernzerhof (PBE) functional,^38^ a generalized gradient approximation, see eqs S7–S10. The kinetic energy is described using the Thomas–Fermi–von Weizsäcker (TFvW) functional as in the previous work.^2,3^ In Figure 1, we compare two sets of exchange–correlation functionals, including the PBE functional and the DW functional used in previous work,^3^ in terms of the chemical potential of electrons in metal bulk,
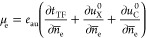
1where *e*_au_ = 27.2 eV is the energy in atomic units, *t*_TF_ is the kinetic energy functional, see eq S6, *u*_X_^0^ is the exchange energy functional of a homogeneous electron gas, eq S8, and *u*_C_^0^ is the correlation energy functional of a homogeneous electron gas, eq S9. *n̅*_e_ is the dimensionless electron density. In this paper, an overbar denotes a dimensionless number density normalized to the reference number density (*a*_0_)^−3^, where *a*_0_ is the reference length, the Bohr radius. It is shown that the chemical energy of electrons μ_e_ increases nearly linearly after a brief decrease as the metal cationic charge density *n̅*_cc_^0^ increases. The difference between these two functionals is within 0.3 eV in the examined range of *n̅*_cc_^0^.^17^ The difference transitions from negative to positive as *n̅*_cc_^0^ increases.

Following Shandilya, Schwarz, and Sundararaman,^39^ we use Morse potentials, expressed in eq S12, instead of truncated power potentials in our previous work,^2,3^ to describe metal–solution interactions. A Morse potential contains three parameters, namely, a well depth *D*_l_, a coefficient controlling the well width β_l_, and an equilibrium bond distance *B*_l_, where the subscript l indexes different solution components (s for solvent, c for cation, a for anion). We are about to show in the following section how these parameters can be determined using first-principles calculations.

Previously, we solved differential equations in terms of *n̅*_e_.^2,3^ It was found that careful treatments are needed for a stable and convergent numerical solution. Specifically, zero or negative *n̅*_e_ must be avoided as *n̅*_e_ occurs in the denominator, and fractional exponents of *n̅*_e_ are required in many places. A positive-defined *n̅*_e_, *n̅*_e_^mod^ = |*n̅*_e_| + *n̅*_e_^lb^ with *n̅*_e_^lb^ being a positive lower bound was used for this end. In this work, we propose a new numerical scheme in which we solve for ψ = (*n̅*_e_)^1/3^ rather than *n̅*_e_. This way, we can get rid of issues related to fractional exponents of *n̅*_e_. The new controlling equation in terms of ψ is given in eq S39.

The modified model is solved in COMSOL Multiphysics. The two controlling equations, eqs S39 and S40, are implemented as a coefficient form partial differential equation. A step-by-step tutorial of constructing the model from scratch is provided in the Supporting Information of this article.

## Model Parameterization and Calibration

A basic set of model parameters are obtained from calibrating the model using experimental data measured on the Ag(111)-KPF_6_ aqueous interface by Valette.^37^ This interface is selected for model calibration for two reasons. First, PF_6_^–^ is found to be a very weakly specifically adsorbing anion on Ag(111), rendering that this interface can well mimic an ideally polarizable interface in a wide potential range that is treated in this model. Specific adsorption and chemisorption of ions were treated using a Newns–Anderson Hamiltonian with several additional parameters in our previous work.^3^ These Hamiltonian parameters can be determined from Kohn–Sham DFT calculations. Calibration of the DPFT model with chemisorption will be reported in a future part of this series. Second, Valette reported *C*_dl_ curves at a series of electrolyte concentrations, allowing us to calibrate the model using a set of *C*_dl_ curves rather than a single one.

The model describes a one-dimensional EDL. The metal cationic cores are represented by a uniform background with a positive charge density *n̅*_cc_^0^, namely,
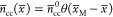
2where  with *N*_Ag_ = 47 representing the number of electrons of a silver atom, and *a*_Ag_ = 4.08 Å is the length of the cubic closed-packed cell of Ag, which contains four silver atoms, and *a*_0_ = 0.529 Å is the Bohr radius. *n̅*_cc_^0^ is normalized to the reference number density (*a*_0_)^−3^. *x̅* = *x*/*a*_0_ is the dimensionless coordinate. This model considers all electrons and does not require pseudopotentials for the metal cationic cores anymore. In an all-electrode model, we should use the vacuum permittivity ϵ_0_ in the Poisson equation for the metal phase.

Within a 1D approximation, we do not consider the discreteness of metal cationic cores, which can be best treated in a three-dimensional (3D) model. We designate the coordinate origin, *x̅* = 0, in the metal bulk phase, and *x̅*_M_ as the dimensionless thickness of the metal region. *x̅*_M_ should be large enough, say 20 (∼1 nm), such that the metal bulk is reached at *x̅* = 0. In the metal bulk, the gradients of electron density and electric potential, denoted ϕ, are zero,
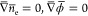
3Here, an overbar denotes variables and operators in the dimensionless system,  with *k*_B_ being the Boltzmann constant, *T*, temperature, *e*_0_, the elementary charge.

In eq 2, θ (*x̅*_M_ – *x̅*) is a Heaviside function, which is equal to 1 when *x̅* < *x̅*_M_ and zero elsewise. The other boundary is designated in the bulk solution, where the boundary conditions are
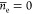
4because metal electrons cannot go beyond several Å from the metal surface, and the electric potential is designated as the reference
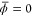
5In this model, the electric potential of the metal, ϕ_M_, is not used explicitly in boundary conditions. Instead, constant-potential simulations of the present model are realized by actually varying μ̃_e_, which is tantamount to varying ϕ_M_ up to a constant of *e*_0_,
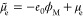
6Note in passing that it is more fundamental to control μ̃_e_ than ϕ_M_ because a voltmeter measures the difference in μ̃_e_, not ϕ_M_ between two electrodes.^40^ We will use −μ̃_e_, which is equivalent to ϕ_M_ up to some constants, to represent electrode potential in the figures of this article. In the subsequent discussion, we will use electrode potential and −μ̃_e_ interchangeably.

After specifying the setup, boundary conditions, and operating conditions of the model, we now explain the acquisition of model parameters. The metal parameters, *n̅*_cc_^0^ and ϵ_op,M_, are already determined for the EDL, and there is no freedom to tune them. We use the PBE functional with recommended gradient coefficients θ_X_ = 0.1235 and θ_C_ = 0.046, and the only free parameter in electronic functionals is the gradient coefficient in the kinetic energy, θ_T_, which is to be determined from calibration with experimental data.

Parameters of the electrolyte solution are adopted from the literature. Molecular dynamics (MD) simulations have determined the radii of hydrated K^+^ and PF_6_^–^ to be within 4–5^41^ and 3–4 Å,^42^ respectively. We use the upper limits, 5 and 4 Å, herein. The length of the cubic lattice accommodating a water molecule is determined to be 3.11 Å, ensuring a bulk concentration of *c*_s_^b^ = 55M. The relative permittivity of the bulk solution is ϵ_b_ = 78.5. Therefore, knowing the optical dielectric constant ϵ_op,S_ of the electrolyte solution, we can calculate the effective dipole moment of water molecules to be  where *k*_B_ is the Boltzmann constant, *T* is the temperature, *and N*_A_ is the Avogadro constant. The only free parameter of the electrolyte solution is thus *ϵ*_op,S_, which is to be determined from calibration with experimental data. The continuous transition of the dielectric constant from ϵ_op,M_ = 1 in the metal phase to *ϵ*_op,S_ in the solution phase is empirically described as
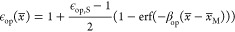
7with a coefficient β_op_ controlling the width of the transition region, and erf (*x̅*) is the error function.

Parameters of the Morse potentials describing metal–solution interactions can be acquired from Kohn–Sham DFT calculations. Water adsorption on Ag(111) has been studied by Le et al. using ab initio molecular dynamics (AIMD).^43^ The binding energy of water is 0.25 eV with a bond length of 2.82 Å. So, the Morse potential of water reads *w*_s_(*x̅*) = *D*_s_(exp(−2β_s_(*x̅* – *B*_s_)) – 2 exp(−β_s_(*x̅* – *B*_s_))) with *D*_s_ = 0.25 eV and . The coefficient β_s_ can be determined by fitting the whole potential profile, which is not available in the AIMD work of Le et al., and we use β_s_ = 1 as an initial guess. Since hydrated K^+^ (subscript c) and PF_6_^–^ (subscript a) are very weakly adsorbing ions, *D*_a_ and *D*_c_ should be much smaller than *D*_s_, while *B*_a_ and *B*_c_ are larger than *B*_s_.

To summarize, unknown model parameters in the current model are θ_T_, ϵ_op,S_, β_l_ (l = s,c,a,op), *D*_l_ (l = c,a), and *B*_l_ (l = c,a). All of these unknown model parameters except θ_T_ can be, in principle, determined from Kohn–Sham DFT calculations. Herein, these unknown model parameters are estimated by comparing model-based and experimental *C*_dl_ curves at five concentrations. The impact of these parameters on the model results will be gauged in a parametric analysis in the next section.

The model calculates *C*_dl_ by differentiating the surface free charge σ_free_ with respect to electrode potential
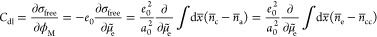
8where the second equal sign transforms the electrode potential to the electrochemical potential of electrons as expressed in eq 6, the third equal sign is due to the definition of , the prefactor  results from the balance of dimensionality, and the fourth equal sign corresponds to another way of calculating σ_free_. These two ways of calculating σ_free_ are equivalent because the total system is electroneutral, ∫d*x̅*(*n̅*_e_ + *n̅*_a_ – *n̅*_c_ – *n̅*_cc_) = 0.

At low concentrations, the *C*_dl_ curve has a Gouy–Chapman minimum that is located at the potential of zero charge (pzc). The specific value of μ̃_e_ corresponding to the pzc, denoted μ̃_e,pzc_, is decomposed into two parts,
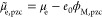
9where ϕ_M,pzc_ is the inner potential of the bulk metal relative to the bulk solution under this condition, and μ_e_ is the chemical potential of electrons in the bulk metal, as expressed in eq 1.

**Figure 2:**
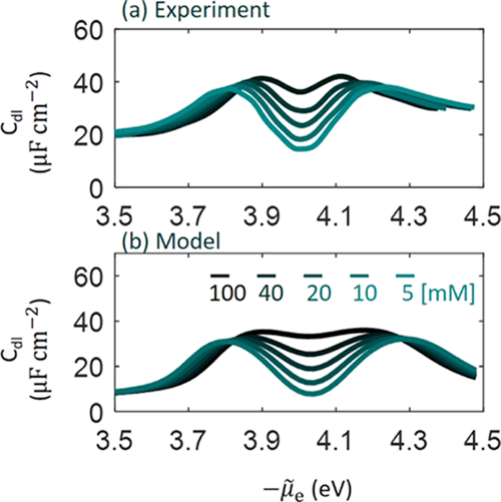
Comparison between model and experimental results of the differential double-layer capacitance (*C*_dl_) of Ag(111)-KPF_6_ aqueous interface at five concentrations indicated in the figure.^37^ Experimental data were reported by Valette in the third part of his fundamental measurements on silver single crystals. Experimental data are corrected using the roughness factor of 1.08 determined by Valette. The model is parameterized according to the experiments. The model results at five concentrations are calculated using a single set of model parameters, which are θ_T_ = 2.08, ϵ_op,S_ = 4, β_l_(l = s,c,a,op) = 1, *D*_l_(l = c,a) = *D*_s_/6, and *B*_l_(l = c,a) = 7.56 corresponding to a bond length of 4 Å.

Figure 2 shows a comparison between model-based and experimental results of *C*_dl_ of Ag(111)-KPF_6_ aqueous interface at five concentrations (100, 40, 20, 10, 5 mM) at 25 ± 2 °C.^37^ The model-based results at five concentrations are calculated using the same set of tunable model parameters θ_T_ = 2.08, ϵ_op,S_ = 4, β_l_(l = s,c,a,op) = 1, *D*_l_(l = c,a) = *D*_s_/6, and *B*_l_(l = c,a) = 7.56. The value of θ_T_ is greater than 5/3 given by the original von Weiszäcker theory, which is not surprising as the latter theory is exact only for a single-electron system.^44^ ϵ_op,S_ falls into the usual range between 3 and 6 for the dielectric constant of the inner layer of the EDL.^45^ When converted to Å^–1^, β_l_ corresponds to 0.53 Å^–1^, which is close to values between 0.4 and 1.1 Å^–1^ for a similar system.^39^*B*_l_ represents a reasonable bond length of 4 Å. The model can well reproduce the well-known camel shape of the *C*_dl_ curve and the lifting trend of the Gouy–Chapman minimum with increasing concentration. Model-based and experimental data are plotted in the same graph in Figure S1, where noticeable differences between them are observed. Experimental values are overall larger than model-based values, which could be attributed to an underestimated roughness factor. The two peaks that signify overcrowding of counterions when the EDL is highly charged are slightly broadened in the model. In addition, model-based values are much lower than experimental values when −μ̃_e_ < 3.8 eV. An improved agreement between the model and experiments can be expected by global optimization of model parameters, which are tuned by hand in this work.

Interestingly enough, the two peaks almost have the same height, though hydrated K^+^ (the left peak) is bigger than PF_6_^–^ (the right peak). This implies that the left peak will be higher than the right one if cations and anions are of the same size, as will be shown later. This asymmetry is ascribed to metal electronic effects in this model. By contrast, without considering metal electronic effects, the classical GCS model with symmetric size would lead to two symmetrical peaks, e.g., see Figure 7 in ref (46). Hatlo, van Roij, and Lue used one size for both cations and anions when fitting their model considering ion polarizability to Valette’s data.^47^ μ̃_e,pzc_ = −4.05 eV, corresponding to a PZC of 0.695 V vs saturated calomel electrode (SCE), is reproduced by the model. The subsequent parametric analysis will show that μ̃_e,pzc_ is mostly affected by θ_T_ and ϵ_op,S_.

**Figure 3:**
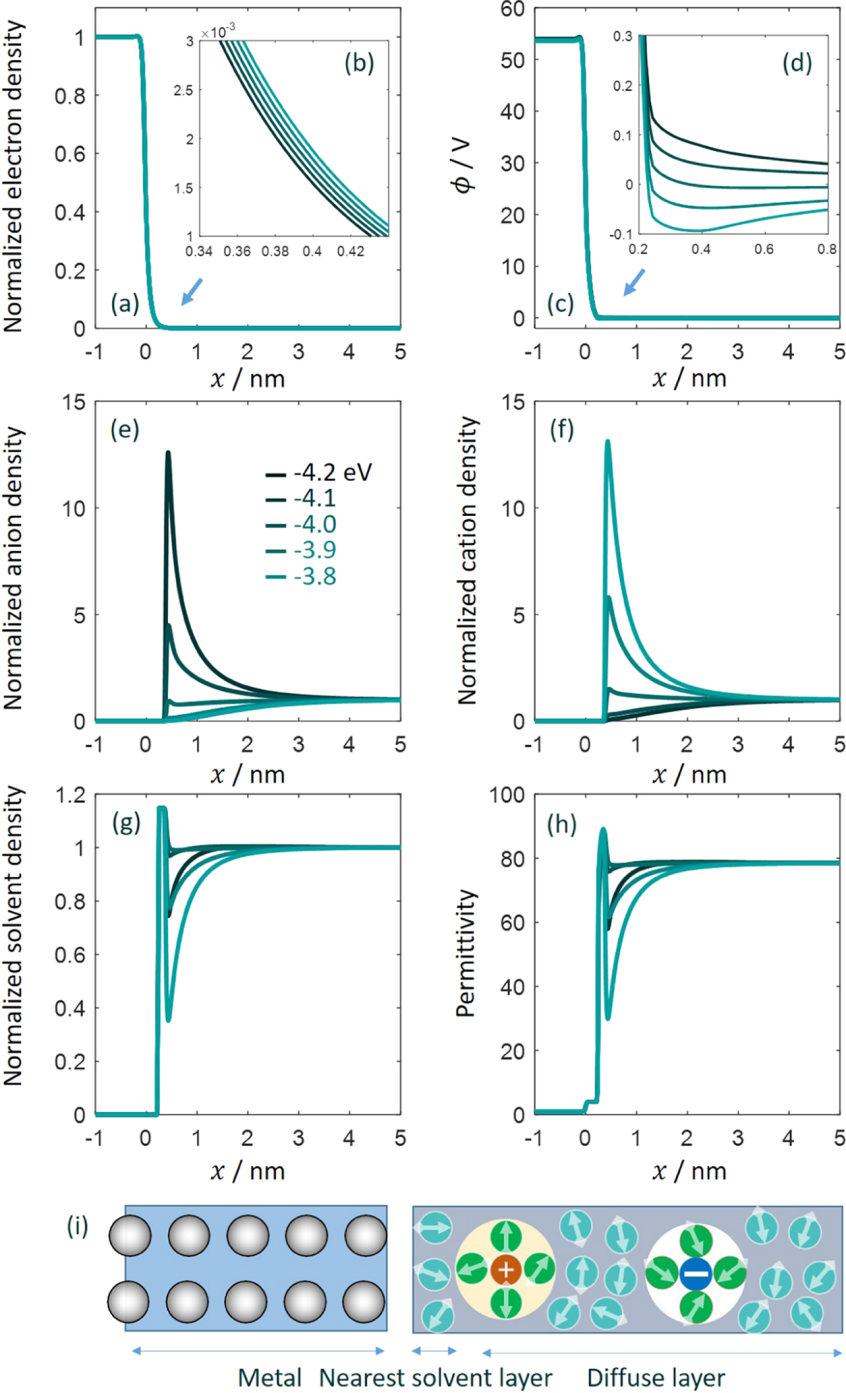
Model results for the Ag(111)-0.1M KPF_6_ aqueous interface at five electrode potentials as indicated in the legends of (e): (a) overall distribution of the electron density normalized to its bulk value, (b) close-up in the spillover region just outside the interface, (c) distribution of electric potential ϕ, (d) close-up just outside the metal, (e) and (f) dimensionless densities of anions and cations normalized to their bulk values, respectively, (g) normalized density of water, and (h) distribution of permittivity. In all of these plots, the metal edge is shifted to *x* = 0. (i) Schematic illustration of the EDL structure with water in the first layer and hydrated ions in the diffuse layer.

The *C*_dl_ curves are overall properties of an EDL. The model can provide spatially resolved, atomistic scale insights of EDL. Figure 3 displays the model results for the Ag(111)-0.1M KPF_6_ aqueous interface at five electrode potentials spanning from negative to positive σ_free_. In these plots, the metal edge is relocated at *x* = 0. Figure 3a shows the overall distribution of the electron density normalized to its bulk value, and (b) gives a close-up of the spillover region just outside the interface. The electron tail stretches out more at lower electrode potentials, *viz*., less negative μ̃_e_ when electrons have a higher potential energy. An overshoot in the electron density is observed near *x* = 0. Figure 3c exhibits the distribution of electric potential ϕ, and a close-up just outside the metal is shown in Figure 3d. A change in the sign of ϕ signifies a change in the sign of σ_free_ and a change in the identity of counterions when varying μ̃_e_. At more negative μ̃_e_, namely, more positive electrode potential, ϕ is more positive, attracting anions into the near-surface region and repelling cations out of the region, as shown in Figure 3e,f for the normalized density of anions and cations, respectively.

All of the above phenomena are already known from classical GCS models^46,48−50^ and in our previous works.^2,3^ The use of a Morse potential that is deeper and closer to the metal surface for water results in a dense packing of water molecules on the metal surface, Figure 3g. Hydrated counterions reside outside of this water layer; see a schematic diagram in Figure 3i. The steric effects of counterions diminish water density beyond the first water layer. The total permittivity is shown in Figure 3h, which follows the distribution of water density. We see a transition from ϵ_op,M_ = 1 in the metal phase to ϵ_op,S_ = 4 around *x* = 2 Å. Even though the densities of water and ions are zero around *x* = 2 Å, ϵ_op,S_ is larger than 1 to account for the screening effects of the electronic cloud of the first water layer.

## Parametric Analysis

We now use the calibrated model to explore the influence of selected model parameters. The parametric analysis will be conducted in three groups. The first group contains parameters related to the electronic structure, including gradient coefficients in electronic functionals, θ_T_ and θ_XC_, and the metal cationic charge density *n̅*_cc_^0^. The second group focuses on the influence of solvent, including the distribution of optical permittivity near the metal surface, described by ϵ_op_(*x̅*) in eq 7, the dipole moment of solvent, and the Morse potential of metal–solvent interactions. The last group consists of parameters related to ions, including ion size, ion concentration, and parameters in Morse potentials of metal–ion interactions.

In addition to *C*_dl_ defined in eq 8, the moment of the electron density distribution will be used in the subsequent analysis, defined as
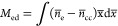
10where *n̅*_cc_ is given by eq 2. A more positive *M*_ed_ represents a more extended distribution of electron density, equivalently, a larger potential drop across the metal surface if the permittivity distribution is fixed.

### Electronic Structure Parameters

**Figure 4:**
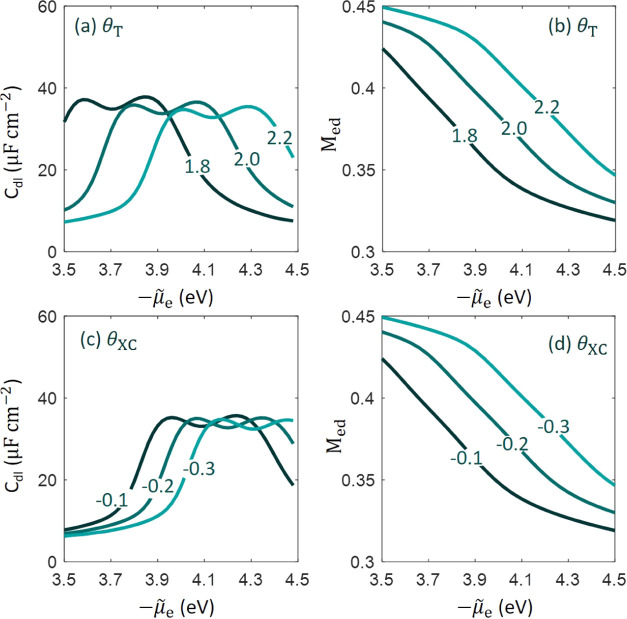
Differential double-layer capacitance (*C*_dl_) curve as a function of the electrochemical potential of electrons, −μ̃_e_, which can be transformed to the electrode potential up to a constant. The two gradient coefficients, θ_T_ in panels (a) and (b), θ_XC_ in panels (c) and (d) are varied at three levels. Effect of θ_T_ and θ_XC_ on the moment of the electron density distribution defined in eq 10 is shown in panels (b) and (d), respectively. In this one-factor-at-a-time study, other parameters except the one under evaluation have their base values.

Figure 4a,c shows the *C*_dl_ curve as a function of −μ̃_e_ at three values of θ_T_ and θ_XC_, respectively. Other parameters except the one under evaluation have their base values in this one-factor-at-a-time study. According to eq 6, varying −μ̃_e_ is equivalent to varying the inner potential of the electrode ϕ_M_. We do not analyze θ_X_ and θ_C_ independently because the gradient terms in the exchange and correlation functionals are combined into one term with one composite gradient coefficient θ_XC_ = θ_X_ – π^2^θ_C_/3.

The *C*_dl_ curves are shifted along the −μ̃_e_ axis with varying θ_T_ and θ_XC_. Specifically, the *C*_dl_ curve is shifted to the right side at more positive θ_T_ and more negative θ_XC_. Accordingly, the pzc is more positive at more positive θ_T_ and more negative θ_XC_. As the chemical potential of electrons μ_e_ is independent of θ_T_ and θ_XC_, as seen from eq 1, variation in the pzc is solely due to variation in ϕ_M,pzc_, according to eq 9, which is determined by the distribution of the electron density at the metal surface.

Figure 4b,d shows the dimensionless moment of the electron density distribution, *M*_ed_, defined in eq 10. The general trend is that *M*_ed_ decreases with more positive electrode potential. That is to say, the electron tail is shrunk at more positive electrode potential when the electrochemical potential of electrons is lower. In addition, *M*_ed_ increases at more positive θ_T_ and more negative θ_XC_. The opposite influence of θ_T_ and θ_XC_ is the consequence of opposite signs of the kinetic and exchange–correlation energy of a homogeneous electron gas. The electron density is distributed in such a profile that the total energy is lowest. The gradient terms in the total energy grow at more positive θ_T_ and more negative θ_XC_ if the electron density distribution is kept the same. To counteract this increasing trend, the electron density distribution should become more even, namely, *M*_ed_ increases.

**Figure 5:**
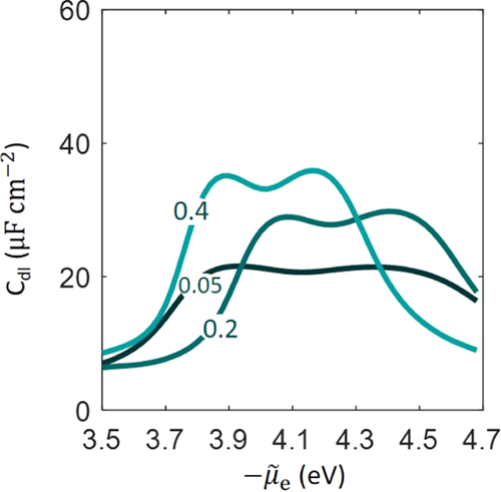
Influence of metal cationic charge density (*n̅*_cc_^0^) on differential double-layer capacitance (*C*_dl_) curve as a function of the electrochemical potential of electrons, −μ̃_e_. Other model parameters have their base values.

Different metals and different facets of a metal have different values of *n̅*_cc_^0^. Figure 5 shows that the *C*_dl_ curve is elevated at larger *n̅*_cc_^0^ because there are more electrons to screen the electric field in the double layer. A nonmonotonic dependence of μ̃_e,pzc_ on *n̅*_cc_^0^ is observed in Figure 5. According to eq 9, μ̃_e,pzc_ is the difference between the chemical part μ_e_ and the electrostatic part e_0_ϕ_M,pzc_. The chemical potential μ_e_ grows with *n̅*_cc_^0^ in the examined range, as shown in Figure 1. ϕ_M,pzc_ also grows with *n̅*_cc_^0^. Therefore, the nonmonotonic dependence of μ̃_e,pzc_ results from the balance of two monotonically increasing trends. It is important to understand that we have used the same θ_T_. At this level of theory, θ_T_ is an empirical parameter that should be calibrated for each metal. In addition, it is about to show that μ̃_e,pzc_ is markedly impacted by ϵ_op_(*x̅*), which depends on the solvent properties.

### Effect of Solvent Properties

**Figure 6:**
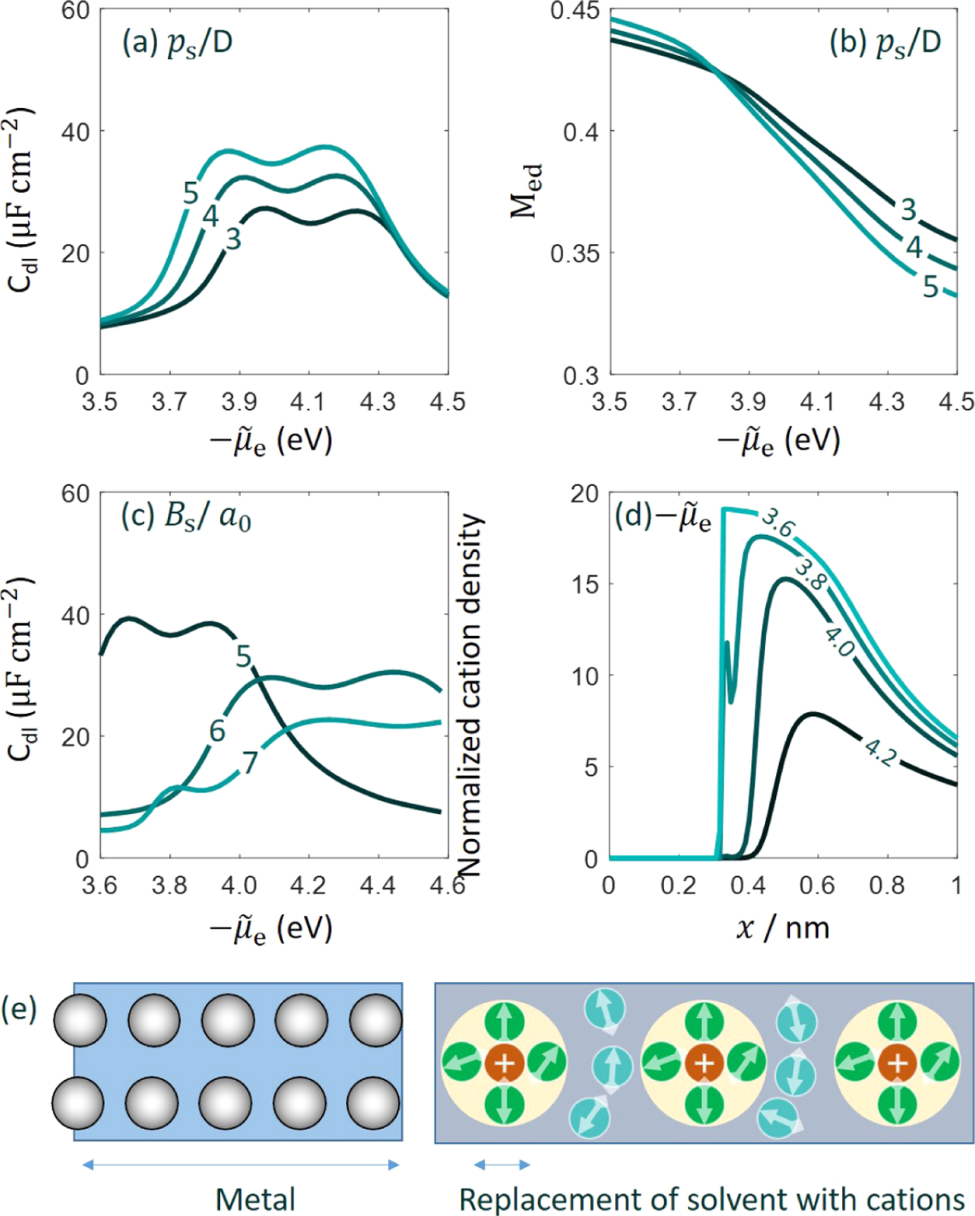
Effect of solvent properties on (a), (c) the differential double-layer capacitance (*C*_dl_), (b) the dimensionless moment of electron density distribution (*M*_ed_), and (d) normalized cation density. Graphs (a) and (b) correspond to the effects of solvent dipole moment *p*_s_, (c) and (d) the metal-bond length in the Morse potential *B*_s_. The line markers in panel (d) represents −μ̃_e_ for the case of *B*_s_ = 7*a*_0_. Panel (e) depicts the scenario where cations as the counterions break into the first solvent layer.

Figure 6 exhibits the influence of solvent parameters, including the solvent dipole moment (*p*_s_) in Figure 6a,b, and the metal–solvent bond length in the Morse potential *B*_s_ in Figure 6c,d. The *C*_dl_ curve is elevated up with increasing *p*_s_ in the unit of Debye (*D*), as expected. In addition, the pzc shifts from −4.1 eV at *p*_s_ = 3D to −4.06 eV at *p*_s_ = 4D and further to −4.02 eV at *p*_s_ = 5D. At larger *p*_s_, Figure 6b shows that the electron density distribution is shrunk when μ̃_e_ is more negative than −3.8 eV but expanded when μ̃_e_ is more positive than −3.8 eV. Overall, *M*_ed_ drops faster with increasing electrode potential when *p*_s_ is higher.

At high electrode potentials, ϕ is positive near the metal surface, see Figure 3d. Solvent with larger *p*_s_ can screen the electric field more efficiently, leading to a less positive ϕ in the electron tail region. This means that chemical potential μ_e_ is higher at a fixed μ̃_e_ when *p*_s_ is larger. According to Figure 1a, μ_e_ decreases with electron density in the electron tail with *n*_e_ < 0.003. Therefore, when *p*_s_ is larger, *n*_e_ should decrease to compensate for the decreased ϕ in the electron tail region at a fixed μ̃_e_.

*C*_dl_ grows when the metal–solvent bond length in the Morse potential *B*_s_ is shortened, as shown in Figure 6c. This phenomenon can be understood readily using Helmholtz’s model of EDL. The capacitance of a planar capacitor is larger when the gap between two end plates constituting the planar capacitor is narrower. When *B*_s_ is increased to 7*a*_0_, namely, when the bond length is similar for solvent and ions, we find a spike of the cation density distribution around 0.35 nm before the metal surface at μ̃_e_ = −3.8 eV, Figure 6d. This spike indicates that cations as the counterions can break into the first solvent layer when the local electric potential is negative enough, as schematically shown in Figure 6e. Such a competition between solvent molecules and counterions was also revealed in our previous work.^3^

Shatla et al. recently studied the pzc of the Au(111)-nonaqueous solution interfaces.^51^ Their study shows that the pzc is 0.31 V_Ag/Ag^+^_ in DMSO with *p*_s_ = 1.97*D*, −0.01 V_Ag/Ag^+^_ in DMSO with *p*_s_ = 3.96*D*, and −0.1 V_Ag/Ag^+^_ in PC with *p*_s_ = 4.9*D*. This trend that the pzc shifts to negative values when *p*_s_ increases is consistent with the model results in Figure 6a. However, the pzc of Ag(111)-ACN aqueous interface with *p*_s_ = 3.93*D* is 0.24 V_Ag/Ag^+^_, which breaks the above trend.

**Figure 7:**
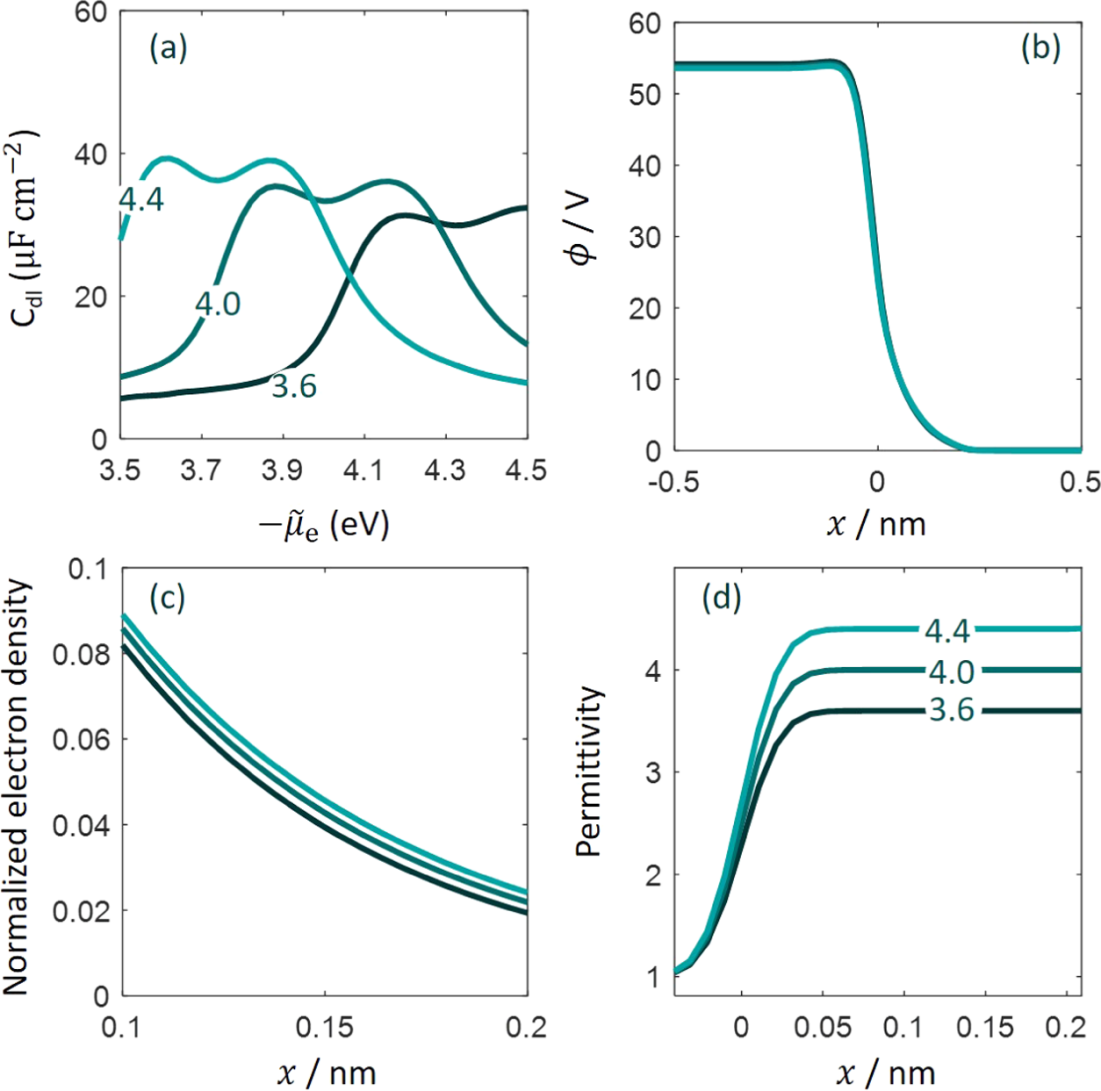
(a) Effect of optical permittivity ϵ_op,S_ on the differential double-layer capacitance (*C*_dl_). Panels (b) and (c) show the distribution of electric potential ϕ and normalized electron density, respectively, at the potential of zero charge. Panel (d) shows the distribution of total permittivity at the potential of zero charge. Plateaus at different values (3.6, 4.0, 4.4) are seen near the metal surface, followed by a transition to the bulk permittivity (not shown). Lines of the same color have the same value of ϵ_op,S_. Other parameters have their basic values.

It is important to note that the model results in Figure 6a are obtained by only changing *p*_s_ while keeping all other parameters unchanged. In experiments, when one changes the solvent, many parameters change at the same time. Therefore, the discrepancy between the model and experiments shall not be surprising. For example, we show in Figure 7 that the pzc can be markedly influenced by other parameters related to the solvent. We change ϵ_op,S_ in eq 7 and keep all other parameters unchanged. Figure 7a shows that the *C*_dl_ curve is elevated, and the pzc decreases when ϵ_op,S_ is increased from 3.6 to 4.4. The total permittivity is shown in Figure 7d. When ϵ_op,S_ is lower, the potential drop across the metal surface at the pzc is more positive, as shown in Figure 7b. Since the potential in the solution bulk is taken as the reference, the inner potential in the metal bulk is higher when ϵ_op,S_ is lower, Figure 7b. A higher inner potential of the metal leads to a more negative μ̃_e,pzc_, namely, a more positive pzc, since the chemical potential μ_e_ in the metal bulk does not change with ϵ_op,S_. As μ̃_e,pzc_ is less negative at larger ϵ_op,S_, electron distribution is more extended outside the metal, Figure 7c.

### Effect of Ion Properties

**Figure 8:**
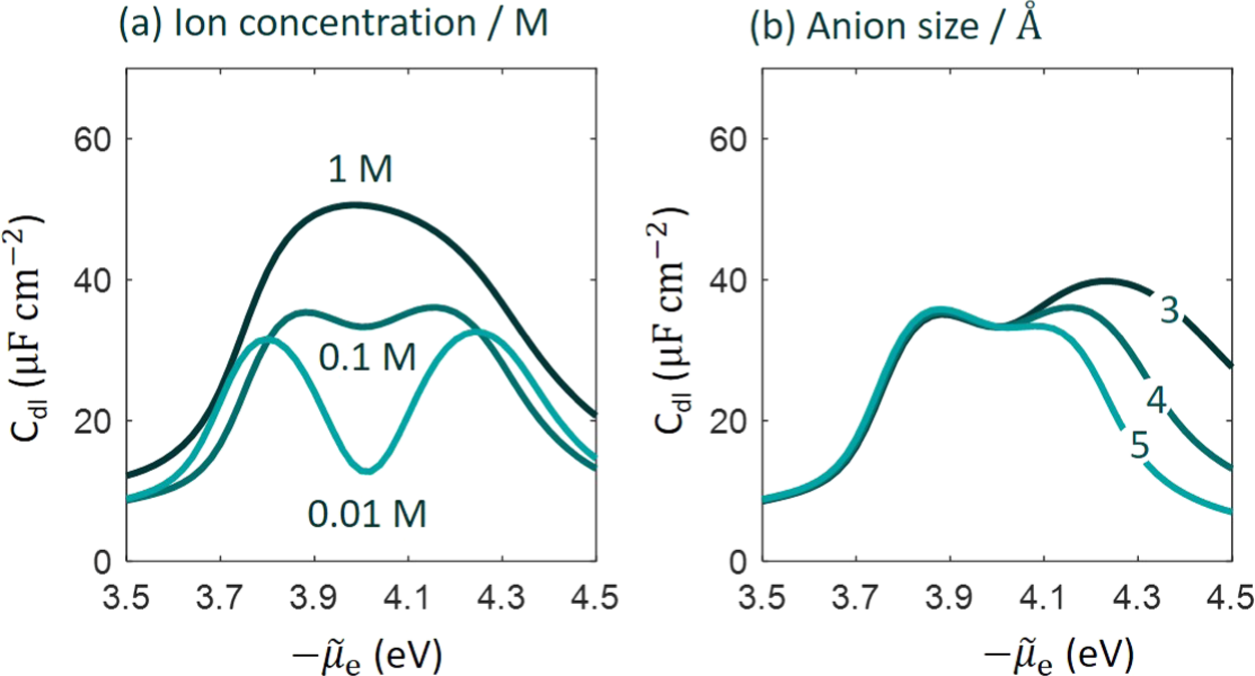
Influence of ion concentration and ion size on differential double-layer capacitance (*C*_dl_) curve as a function of the electrochemical potential of electrons, μ̃_e_. In panel (a), the radius of cations and anions are 5 and 4 Å, respectively, as in the base case. In panel (b), we vary the anion radius from 3 to 5 Å.

The ion concentration is one of the easiest parameters tunable in experiments. In Figure 8, we examine how the *C*_dl_ curve depends on the ion concentration while all other parameters have their base values. Compared to Figure 2, we include a more concentrated electrolyte solution of 1M. The camel shape with the minima at the pzc is transitioned to a volcano shape when the ion concentration increases. The camel–volcano transition has been clearly exposited by Kornyshev,^48^ and many others; see a recent review.^49^ However, these classical EDL models would predict the volcano peak is also at the pzc where the camel minima are located. By contrast, the present model shows a left shift of the volcano peak relative to the pzc, which is a manifestation of metal electronic effects.

The model uses different sizes for cations and anions. Specifically, the radius of hydrated K^+^ and PF_6_^–^ are 5 and 4 Å, respectively. According to classical EDL models,^46,48−50^ the size asymmetry will lead to asymmetric camel shapes. For the present case, one would expect that the peak at lower electrode potential that signifies crowding of larger cations would be smaller than that at more positive electrode potential. By contrast, experimental data and model results show a nearly symmetric camel shape. In Figure 8b, we show the model results when the anion radius varies between 3, 4, and 5 Å. The peak at more positive electrode potential grows when the anion radius is smaller. When cations and anions have the same radius of 5 Å, the peak at lower electrode potential is higher, which would not be expected from classical EDL models. This again reflects the essential role of metal electrons. At lower electrode potentials, electron density is extended more outside the metal, see Figure 3b, enhancing the capability of screening the electric field emanated from the metal, namely, increasing the *C*_dl_.

**Figure 9:**
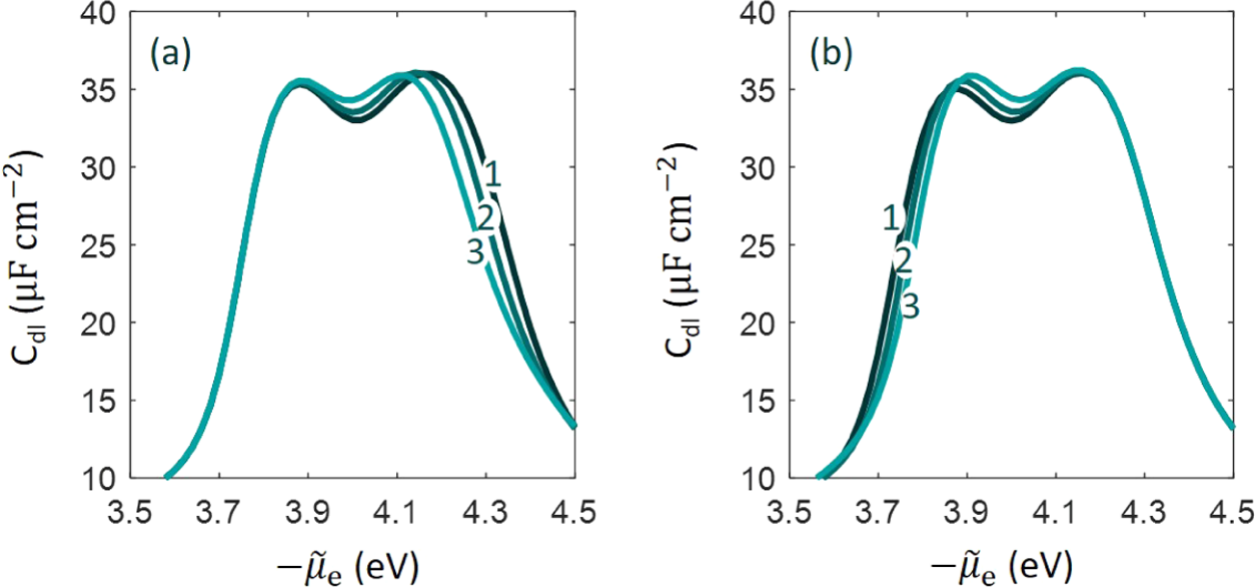
Influence of the well depth (a) *D*_a_ (dimensionless value normalized to *k*_B_*T*) and (b) *D*_c_ (dimensionless value normalized to *k*_B_*T*) on the differential double-layer capacitance curve as a function of electrochemical potential of electrons, μ̃_e_.

The influence of the well depth of Morse potential on *C*_dl_ is examined in Figure 9a,b for anions and cations, respectively. The anion effect is seen on the right peak at more positive electrode potentials when the metal surface is positively charged, while the cation effect is seen on the left side at more negative electrode potentials when the metal surface is negatively charged. Around the pzc, *C*_dl_ grows when the well depth is larger for both cations and anions because it effectively enriches the local concentration. This has been recently discussed in detail by Doblhoff–Dier and Koper in the context of the EDL at Pt-aqueous solution interfaces.^52^ A shift in the pzc is observed for both cases when the well depth is changed. This reminds us of the fact that the pzc is an interfacial property that is determined by both parties constituting the interface, namely, the metal and the nearby electrolyte solution. A change in the local reaction environment in the EDL would change the pzc, even though the cations and anions are not specifically adsorbed. Note in passing that Valette used the shift in the pzc at different ion concentrations as a descriptor of ion-specific adsorption.^53−55^ The model results presented in Figure 9 reveal that it might be problematic to use the concentration-dependent shift of the pzc as a descriptor of specific adsorption.

## Conclusions

We have modified the density-potential functional theory (DPFT) of electrochemical interfaces using a generalized gradient approximation for exchange–correlation energy and Morse potentials for metal–solvent/ions interactions. We have calibrated an EDL model based on DPFT using experimental differential double-layer capacitance (*C*_dl_) curves measured on Ag(111)-KPF_6_ aqueous solution by Valette. The calibrated model has then been subject to a parametric study in which parameters of metal electrons, solvent, and ions are examined.

The difference in chemical potential of electrons between the PBE functional and the Dirac–Wagner functional is within 0.3 eV when the charge density of metal cationic cores (*n̅*_cc_^0^) varies from 10^–4^ to 10^–1^. The difference in work function and potential of zero charge is thus mainly due to the interfacial potential drop and the electron density distribution across the metal surface. In this regard, the gradient coefficients in kinetic and exchange–correlation functionals, namely, θ_T_ and θ_XC_, as well as the permittivity near the metal surface, characterized by ϵ_op,S_, are important. A larger θ_T_, a more negative θ_XC_, and a lower ϵ_op,S_ lead to a more extended metal electron tail, resulting in a larger interfacial potential drop and a more positive potential of zero charge (pzc). *C*_dl_ at the pzc is larger when the charge density of metal cationic cores (*n̅*_cc_^0^) is larger. However, the pzc varies nonmonotonically with *n̅*_cc_^0^. The *C*_dl_ curve is elevated, while the pzc decreases when the solvent dipole moment (*p*_s_) is greater. Changing the well depth of Morse potential affects different peaks of the *C*_dl_ curve for cations and anions and also shifts the pzc. The parametric analysis indicates that θ_T_, the permittivity within 1 nm from the metal surface, and metal–solution interactions are key parameters of the present model, which should be calibrated carefully with first-principles methods and experiments.

This study also reveals several aspects of the difference between the present model and classical EDL models. In terms of physics, the present model contains quantum mechanical effects of metal electrons, as well as short-range interactions between the metal and solvent/ions; both are not considered in many classical EDL models. This allows this model to calculate the pzc, which is usually taken as a potential reference in classical EDL models. In terms of phenomenology, the present model and classical EDL models differ in describing the dependence of *C*_dl_ on the ion size and concentration. Both models give a camel–volcano transition of the shape of *C*_dl_ with increasing the ion concentration. Additionally, the present model shows that the peak of the volcano peak at high ion concentration does not coincide with the minimum of the camel-shaped *C*_dl_. Classical EDL models would lead to a camel-shaped *C*_dl_ curve with two symmetric peaks when cations and anions have the same size. However, the present model gives a camel-shaped *C*_dl_ curve with a higher left peak for the case of symmetric size. Both discrepancies between this model and EDL models are attributed to metal electronic effects.

## Supporting Information Available

Theory; grand potential functional; variational analysis; formal transformation; more details about numerical implementation; initial guesses; tips for numerical stability and convergence; and Figures S1–S7 (PDF)

## References

[ref1] Huang J.; Li P.; Chen S. Potential of Zero Charge and Surface Charging Relation of Metal-Solution Interphases from a Constant-Potential Jellium-Poisson-Boltzmann Model. Phys. Rev. B 2020, 101, 125422 10.1103/PhysRevB.101.125422.

[ref2] Huang J. Hybrid Density-Potential Functional Theory of Electric Double Layers. Electrochim. Acta 2021, 389, 138720 10.1016/j.electacta.2021.138720.

[ref3] Huang J.; Chen S.; Eikerling M. Grand-Canonical Model of Electrochemical Double Layers from a Hybrid Densitypotential Functional. J. Chem. Theory Comput. 2021, 17, 2417–2430. 10.1021/acs.jctc.1c00098. 33787259

[ref4] Jinnouchi R.; Anderson A. B. Electronic Structure Calculations of Liquid-Solid Interfaces: Combination of Density Functional Theory and Modified Poisson-Boltzmann Theory. Phys. Rev. B 2008, 77, 245417 10.1103/PhysRevB.77.245417.

[ref5] Letchworth-Weaver K.; Arias T. A. Joint Density Functional Theory of the Electrode-Electrolyte Interface: Application to Fixed Electrode Potentials, Interfacial Capacitances, and Potentials of Zero Charge. Phys. Rev. B 2012, 86, 075140 10.1103/PhysRevB.86.075140.

[ref6] Sundararaman R.; Goddard W. A.; Arias T. A. Grand Canonical Electronic Density-Functional Theory: Algorithms and Applications to Electrochemistry. J. Chem. Phys. 2017, 146, 114104 10.1063/1.4978411. 28330356

[ref7] Melander M. M.; Kuisma M. J.; Christensen T. E. K.; Honkala K. Grand-Canonical Approach to Density Functional Theory of Electrocatalytic Systems: Thermodynamics of Solid-Liquid Interfaces at Constant Ion and Electrode Potentials. J. Chem. Phys. 2018, 150, 041706. 10.1063/1.5047829 30709274

[ref8] Hörmann N. G.; Andreussi O.; Marzari N. Grand Canonical Simulations of Electrochemical Interfaces in Implicit Solvation Models. J. Chem. Phys. 2019, 150, 041730 10.1063/1.5054580. 30709280

[ref9] Mathew K.; Kolluru V. S. C.; Mula S.; Steinmann S. N.; Hennig R. G. Implicit Self-Consistent Electrolyte Model in Plane-Wave Density-Functional Theory. J. Chem. Phys. 2019, 151, 234101 10.1063/1.5132354. 31864239

[ref10] Nishihara S.; Otani M. Hybrid Solvation Models for Bulk, Interface, and Membrane: Reference Interaction Site Methods Coupled with Density Functional Theory. Phys. Rev. B 2017, 96, 115429 10.1103/PhysRevB.96.115429.

[ref11] Fernandez-Alvarez V. M.; Eikerling M. H. Interface Properties of the Partially Oxidized Pt(111) Surface Using Hybrid Dft–Solvation Models. ACS Appl. Mater. Interfaces 2019, 11, 43774–43780. 10.1021/acsami.9b16326. 31650835

[ref12] Tesch R.; Kowalski P. M.; Eikerling M. H. Properties of the Pt(111)/Electrolyte Electrochemical Interface Studied with a Hybrid Dft–Solvation Approach. J. Phys.: Condensed Matter 2021, 33, 444004 10.1088/1361-648X/ac1aa2. 34348250

[ref13] Kovalenko A.; Hirata F. Self-Consistent, Kohn-Sham Dft and Three-Dimensional Rism Description of a Metal-Molecular Liquid Interface. J. Mol. Liq. 2001, 90, 215–224. 10.1016/S0167-7322(01)00124-6.

[ref14] Wang Y. A.; Carter E. A. Orbital-Free Kinetic-Energy Density Functional Theory. In Theoretical Methods in Condensed Phase Chemistry, Schwartz S. D., Ed.; Springer Netherlands: Dordrecht, 2002; pp 117–184.

[ref15] Witt W. C.; del Rio B. G.; Dieterich J. M.; Carter E. A. Orbital-Free Density Functional Theory for Materials Research. J. Mater. Res. 2018, 33, 777–795. 10.1557/jmr.2017.462.

[ref16] Lieb E. H. Thomas-Fermi and Related Theories of Atoms and Molecules. In The Stability of Matter: From Atoms to Stars: Selecta of Elliott H. Lieb, Thirring W., Ed.; Springer: Berlin, Heidelberg, 1997; pp 259–297.

[ref17] Smith J. R. Self-Consistent Many-Electron Theory of Electron Work Functions and Surface Potential Characteristics for Selected Metals. Phys. Rev. 1969, 181, 522–529. 10.1103/PhysRev.181.522.

[ref18] Lang N. D. The Density-Functional Formalism and the Electronic Structure of Metal Surfaces. In Solid State Physics, Ehrenreich H.; Seitz F.; Turnbull D., Eds.; Academic Press, 1974; Vol. 28, pp 225–300.

[ref19] Lang N. D.; Kohn W. Theory of Metal Surfaces: Charge Density and Surface Energy. Phys. Rev. B 1970, 1, 4555–4568. 10.1103/PhysRevB.1.4555.

[ref20] Chen M.; Xia J.; Huang C.; Dieterich J. M.; Hung L.; Shin I.; Carter E. A. Introducing Profess 3.0: An Advanced Program for Orbital-Free Density Functional Theory Molecular Dynamics Simulations. Comput. Phys. Commun. 2015, 190, 228–230. 10.1016/j.cpc.2014.12.021.

[ref21] Ciracì C.; Della Sala F. Quantum Hydrodynamic Theory for Plasmonics: Impact of the Electron Density Tail. Phys. Rev. B 2016, 93, 205405 10.1103/PhysRevB.93.205405.

[ref22] Michta D.; Graziani F.; Bonitz M. Quantum Hydrodynamics for Plasmas—a Thomas-Fermi Theory Perspective. Contrib. Plasma Phys. 2015, 55, 437–443. 10.1002/ctpp.201500024.

[ref23] Moldabekov Z. A.; Bonitz M.; Ramazanov T. S. Theoretical Foundations of Quantum Hydrodynamics for Plasmas. Phys. Plasmas 2018, 25, 031903 10.1063/1.5003910.

[ref24] Xiang H.; Wang Z.; Xu L.; Zhang X.; Lu G. Quantum Plasmonics in Nanorods: A Time-Dependent Orbital-Free Density Functional Theory Study with Thousands of Atoms. J. Phys. Chem. C 2020, 124, 945–951. 10.1021/acs.jpcc.9b10510.

[ref25] Badiali J. P.; Rosinberg M. L.; Goodisman J. Contribution of the Metal to the Differential Capacity of an Ideally Polarisable Electrode. J. Electroanal. Chem. Interfacial Electrochem. 1983, 143, 73–88. 10.1016/S0022-0728(83)80255-1.

[ref26] Badiali J. P.; Rosinberg M. L.; Vericat F.; Blum L. A Microscopic Model for the Liquid Metal-Ionic Solution Interface. J. Electroanal. Chem. Interfacial Electrochem. 1983, 158, 253–267. 10.1016/S0022-0728(83)80611-1.

[ref27] Schmickler W. A Jellium-Dipole Model for the Double Layer. J. Electroanal. Chem. Interfacial Electrochem. 1983, 150, 19–24. 10.1016/S0022-0728(83)80185-5.

[ref28] Schmickler W.; Henderson D. The Interphase between Jellium and a Hard Sphere Electrolyte. A Model for the Electric Double Layer. J. Chem. Phys. 1984, 80, 3381–3386. 10.1063/1.447092.

[ref29] Kornyshev A. A. Metal Electrons in the Double Layer Theory. Electrochim. Acta 1989, 34, 1829–1847. 10.1016/0013-4686(89)85070-4.

[ref30] Seino J.; Kageyama R.; Fujinami M.; Ikabata Y.; Nakai H. Semi-Local Machine-Learned Kinetic Energy Density Functional with Third-Order Gradients of Electron Density. J. Chem. Phys. 2018, 148, 241705 10.1063/1.5007230. 29960373

[ref31] Constantin L. A.; Fabiano E.; Della Sala F. Performance of Semilocal Kinetic Energy Functionals for Orbital-Free Density Functional Theory. J. Chem. Theory Comput. 2019, 15, 3044–3055. 10.1021/acs.jctc.9b00183. 30964665

[ref32] Xu Q.; Wang Y.; Ma Y. Nonlocal Kinetic Energy Density Functional Via Line Integrals and Its Application to Orbital-Free Density Functional Theory. Phys. Rev. B 2019, 100, 205132 10.1103/PhysRevB.100.205132.

[ref33] Kalita B.; Li L.; McCarty R. J.; Burke K. Learning to Approximate Density Functionals. Acc. Chem. Res. 2021, 54, 818–826. 10.1021/acs.accounts.0c00742. 33534553

[ref34] Abidi N.; Lim K. R. G.; Seh Z. W.; Steinmann S. N. Atomistic Modeling of Electrocatalysis: Are We There Yet?. Wiley Interdiscip. Rev.: Comput. Mol. Sci. 2020, 11, e1499 10.1002/wcms.1499.

[ref35] Groß A. Theory of Solid/Electrolyte Interfaces. Surf. Interface Sci. 2020, 471–515. 10.1002/9783527680603.ch56.

[ref36] Ringe S.; Hörmann N. G.; Oberhofer H.; Reuter K. Implicit Solvation Methods for Catalysis at Electrified Interfaces. Chem. Rev. 2022, 122, 10777–10820. 10.1021/acs.chemrev.1c00675. 34928131 PMC9227731

[ref37] Valette G. Double Layer on Silver Single Crystal Electrodes in Contact with Electrolytes Having Anions Which Are Slightly Specifically Adsorbed: Part Iii. The (111) Face. J. Electroanal. Chem. 1989, 269, 191–203. 10.1016/0022-0728(89)80112-3.

[ref38] Perdew J. P.; Burke K.; Ernzerhof M. Generalized Gradient Approximation Made Simple. Phys. Rev. Lett. 1996, 77, 3865–3868. 10.1103/PhysRevLett.77.3865. 10062328

[ref39] Shandilya A.; Schwarz K.; Sundararaman R. Interfacial Water Asymmetry at Ideal Electrochemical Interfaces. J. Chem. Phys. 2021, 156, 014705 10.1063/5.0076038. 34998343

[ref40] Boettcher S. W.; Oener S. Z.; Lonergan M. C.; Surendranath Y.; Ardo S.; Brozek C.; Kempler P. A. Potentially Confusing: Potentials in Electrochemistry. ACS Energy Lett. 2021, 6, 261–266. 10.1021/acsenergylett.0c02443.

[ref41] Rowley C. N.; Roux Bt. The Solvation Structure of Na+ and K+ in Liquid Water Determined from High Level Ab Initio Molecular Dynamics Simulations. J. Chem. Theory Comput. 2012, 8, 3526–3535. 10.1021/ct300091w. 26593000

[ref42] Raju S. G.; Balasubramanian S. Aqueous Solution of [Bmim][Pf6]: Ion and Solvent Effects on Structure and Dynamics. J. Phys. Chem. B 2009, 113, 4799–4806. 10.1021/jp8111777. 19338368

[ref43] Le J.; Cuesta A.; Cheng J. The Structure of Metal-Water Interface at the Potential of Zero Charge from Density Functional Theory-Based Molecular Dynamics. J. Electroanal. Chem. 2018, 819, 87–94. 10.1016/j.jelechem.2017.09.002.

[ref44] Weizsäcker C. F. v. Zur Theorie Der Kernmassen. Z. Phys. 1935, 96, 431–458. 10.1007/BF01337700.

[ref45] Bockris J. O. m.; Devanathan M. A. V.; Müller K.; Butler J. A. V. On the Structure of Charged Interfaces. Proc. R. Soc. London, Ser. A 1963, 274, 55–79.

[ref46] Lu-Lu Zhang C.-K. L. J. H. A Beginners’ Guide to Modelling of Electric Double Layer under Equilibrium, Nonequilibrium and Ac Conditions. J. Electrochem. 2022, 28, 2108471 10.13208/j.electrochem.210847.

[ref47] Hatlo M. M.; van Roij R.; Lue L. The Electric Double Layer at High Surface Potentials: The Influence of Excess Ion Polarizability. Europhys. Lett. 2012, 97, 28010 10.1209/0295-5075/97/28010.

[ref48] Kornyshev A. A. Double-Layer in Ionic Liquids: Paradigm Change?. J. Phys. Chem. B 2007, 111, 5545–5557. 10.1021/jp067857o. 17469864

[ref49] Budkov Y. A.; Kolesnikov A. L. Electric Double Layer Theory for Room Temperature Ionic Liquids on Charged Electrodes: Milestones and Prospects. Curr. Opin. Electrochem. 2022, 33, 100931 10.1016/j.coelec.2021.100931.

[ref50] Bazant M. Z.; Kilic M. S.; Storey B. D.; Ajdari A. Towards an Understanding of Induced-Charge Electrokinetics at Large Applied Voltages in Concentrated Solutions. Adv. Colloid Interface Sci. 2009, 152, 48–88. 10.1016/j.cis.2009.10.001. 19879552

[ref51] Shatla A. S.; Landstorfer M.; Baltruschat H. On the Differential Capacitance and Potential of Zero Charge of Au(111) in Some Aprotic Solvents. ChemElectroChem 2021, 8, 1817–1835. 10.1002/celc.202100316.

[ref52] Doblhoff-Dier K.; Koper M. T. M. Modeling the Gouy–Chapman Diffuse Capacitance with Attractive Ion–Surface Interaction. J. Phys. Chem. C 2021, 125, 16664–16673. 10.1021/acs.jpcc.1c02381.

[ref53] Valette G. Double Layer on Silver Single Crystal Electrodes in Contact with Electrolytes Having Anions Which Are Slightly Specifically Adsorbed: Part Ii. The (100) Face. J. Electroanal. Chem. Interfacial Electrochem. 1982, 138, 37–54. 10.1016/0022-0728(82)87126-X.

[ref54] Valette G. Double Layer on Silver Single Crystal Electrodes in Contact with Electrolytes Having Anions Which Are Slightly Specifically Adsorbed: Part Iii. The (111) Face. J. Electroanal. Chem. Interfacial Electrochem. 1989, 269, 191–203. 10.1016/0022-0728(89)80112-3.

[ref55] Valette G. Double Layer on Silver Single-Crystal Electrodes in Contact with Electrolytes Having Anions Which Present a Slight Specific Adsorption: Part I. The (110) Face. J. Electroanal. Chem. Interfacial Electrochem. 1981, 122, 285–297. 10.1016/S0022-0728(81)80159-3.

