# Supplementary material for: Density-Potential Functional Theory of Electrochemical Double Layers: Calibration on the Ag(111)-KPF6 System and Parametric Analysis
Source: J Chem Theory Comput. 2023 Jan 18;19(3):1003–13. doi: 10.1021/acs.jctc.2c00799 (PMC9933430; doi:10.1021/acs.jctc.2c00799)
Supplement: Supplementary file 1 — ct2c00799_si_001.pdf [file ct2c00799_si_001.pdf]

Density-Potential Functional Theory of Electrochemical Double Layers. Calibration on the Ag(111)-KPF_6 system and Parametric Analysis

Jun Huang

In this supplementary document, I supply a detailed derivation of the theory, a figure and its implementation in COMSOL.

Theory

Grand potential functional

The electrode is composed of cationic cores (denoted by the subscript cc in variables) and valence electrons (e). The electrolyte solution is composed of cations (c), anions (a), and solvent molecules (s). Electrons must be treated as quantum mechanical objects, while other particles can be treated as classical charged particles that interact with each other via coulombic forces, hard-sphere repulsion and other short-ranged forces, if any.

Our primary task is to calculate the grand potential Ω of the EDL, which is transformed from the Helmholtz free energy F via,

$$\Omega = F - \int d^3r \left(n_{\mathrm{e}}\tilde{\mu}_{\mathrm{e}} + \sum_{l=1}^{N_{\mathbb{C}}} n_l \tilde{\mu}_l \right), \tag{S1}$$

where n_{e} is the electron density, $\tilde{\mu}_{\mathrm{e}}$ is the electrochemical potential of electrons, the last term is a summation of $N_{\mathbb{C}}$ types of charged particles in solution, $\tilde{\mu}_l$ is the electrochemical potential of charged particles of type l. The last two terms mean that the EDL is connected with an electron reservoir on one side and an electrolyte solution reservoir on the other side.

The first ansatz says that the total Helmholtz free energy can be divided into a quantum mechanical part, a classical part, and their interactions,

$$F = F_{\mathbb{Q}} + F_{\mathbb{C}} + F_{\otimes}, \tag{S2}$$

where $F_{\mathbb{Q}}$ describes the quantum-mechanical behavior of the electron gas, $F_{\mathbb{C}}$ describes classical behavior of charged particles, and $F_{\otimes}$ describes interactions between the electron gas and classical charged particles.

The task of formulating the classical part, $F_{\mathbb{C}}$, is essentially to obtain the Gibbs free energy of a grand-canonical inhomogeneous fluid of charged particles that interact via not only long-range coulombic interactions, but also short-range interactions, *e.g.*, the hard-sphere repulsion. In a previous work,[1] the complexity is coped with by introducing a reference system which accounts for two-particle interactions except the coulombic interaction and whose properties are supposed to be known a prior. This treatment leads to,

$$F_{\mathbb{C}} = \int d^3r\, f_{\mathbb{C}}, \tag{S3}$$

where $f_{\mathbb{C}}$ is the volumetric Helmholtz free energy,

$$f_{\mathbb{C}} = -\frac{1}{2}\epsilon_{\mathrm{op}}(\nabla\phi)^2 + \sum_{l=1}^{N_{\mathbb{C}}} n_l\left(\delta(l\in M)q_l\phi - \delta(l\in S)\beta^{-1}\ln\frac{\sinh\left(\beta p_l|\nabla\phi|\right)}{\beta p_l|\nabla\phi|}\right)$$
$$+\sum_{l=1}^{N_{\mathbb{C}}}\beta^{-1}n_l\left(\ln\left(n_l\Lambda_l^3\right)-1\right) + \Phi_{\mathrm{ex}}(\{\rho_l\}) + (n_{\mathrm{cc}} - n_{\mathrm{e}})e_0\phi, \tag{S4}$$

Here, the first term on the right hand side (RHS) represents the self-energy of the electric field, which is obtained from Hubbard–Stratonovich transformation of particle-particle interactions, with ϵ_{op} the permittivity, ϕ the electric potential. Notice that ϵ_{op} usually varies spatially because the electrode and the electrolyte can have different permittivities, denoted by $\epsilon_{\mathrm{op,M}}$ and $\epsilon_{\mathrm{op,S}}$, respectively. Since we have considered the orientational polarization of solvent, $\epsilon_{\mathrm{op,S}}$ accounts mainly for electronic polarization of ions and solvent molecules. In the metal phase, all electrons are included in the theory and therefore, $\epsilon_{\mathrm{op,M}}$ is nothing but the vacuum permittivity. The second term is the potential energies of charged particles in solution due to particle-particle coulombic interactions, with $\beta = (k_{\mathrm{B}}T)^{-1}$ the inverse thermal energy, p_l the dipole moment. The symbol $\delta(l\in M)$ is equal to one for monopolar (M) charged particles, *e.g.*, cations

and anions, and zero otherwise, $\delta(l \in S)$ is equal to one for dipolar solvent molecules (S) and zero otherwise. The third term represents the Gibbs free energy of an ideal-gas reference system, where Λ_l is the thermal wavelength of particle l. The fourth term $\Phi_{\text{ex}}(\{n_l\})$ accounts for excess Gibbs free energy when the reference system deviates from an ideal gas system.[2] The fifth term represents the Hartree energy of electrons and cationic cores of the electrode.

$F_{\mathbb{Q}}$ is where the theory of inhomogeneous electron gas enters in the current framework.[3-4] According to the Kohn-Sham scheme,[5] we divide $F_{\mathbb{Q}}$ as follows,

$$F_{\mathbb{Q}} = T_{\text{ni}}[n_e,\nabla n_e,\ldots] + U_{\text{XC}}[n_e,\nabla n_e,\ldots], \tag{S5}$$

where $T_{\text{ni}}[n_e,\nabla n_e,\ldots]$ is the kinetic energy of electrons, $U_{\text{XC}}[n_e,\nabla n_e,\ldots]$ is the exchange-correlation energy. The entropy of the electron gas is implicitly considered in the parameters of electronic functionals as in ref.[6].

T_{ni} is described by the Thomas-Fermi-von Weizsäcker theory,[3, 7-8]

$$T_{\text{ni}} = \int d^3r\, t_{\text{ni}} = \int d^3r\, e_{\text{au}} a_0^{-3} t_{\text{TF}}(1 + \theta_{\text{T}} s^2), \tag{S6}$$

where t_{ni} is the volumetric kinetic energy, t_{TF} is the volumetric kinetic energy given by the Thomas-Fermi theory, $t_{\text{TF}} = \frac{3}{10}(3\pi^2)^{\frac{2}{3}}(n_e a_0^3)^{\frac{5}{3}}$, $(1 + \theta_T s^2)$ is the correction for gradient terms, $s = {|\nabla n_{\text{e}}|} \Big/ \left(2(3\pi^2)^{\frac{1}{3}}(n_{\text{e}})^{\frac{4}{3}}\right)$ is the reduced gradient term, and θ_T is a gradient coefficient tuning the contribution of the gradient term. The term $e_{\text{au}} a_0^{-3}$ is used to transform the expression from atomic units to SI units, with $e_{\text{au}} = e_0^2/(4\pi\epsilon_0 a_0)$ the atomic energy, a_0 the Bohr radius, e_0 the unit of electrical charge, and ϵ_0 the vacuum permittivity.

U_{XC} is the sum of an exchange part u_{X} and a correlation part u_{C},[3]

$$U_{\mathrm{XC}} = \int d^3r\,(u_{\mathrm{X}} + u_{\mathrm{C}}), \tag{S7}$$

where u_{X} is expanded as,

$$u_{\mathrm{X}} = e_{\mathrm{au}} a_0^{-3} u_{\mathrm{X}}^0 (1 + \theta_{\mathrm{X}} s^2), \tag{S8}$$

with the volumetric exchange energy of a uniform electron gas, $u_{\mathrm{X}}^0 = -\frac{3}{4}\left(\frac{3}{\pi}\right)^{\frac{1}{3}}(n_{\mathrm{e}} a_0^3)^{\frac{4}{3}}$, θ_{X} is a gradient coefficient tuning the contribution of the gradient term in the exchange energy. Similarly, u_{C} is expanded as

$$u_{\mathrm{C}} = e_{\mathrm{au}} a_0^{-3} (u_{\mathrm{C}}^0 + \theta_{\mathrm{C}} n_e a_0^3 t^2), \tag{S9}$$

where $t = a_0^4 |\nabla n_{\mathrm{e}}| \Big/ \left(4\left(\frac{3}{\pi}\right)^{\frac{1}{6}} (n_{\mathrm{e}} a_0^3)^{\frac{7}{6}}\right)$ is another reduced density gradient in terms of dimensional n_{e} and coordinates, and u_{C}^0 is the volumetric correlation energy of a uniform electron gas, for which we use the interpolation relation of Perdew et al.,[4]

$$u_{\mathrm{C}}^0 = -2\alpha_1 n_e a_0^3 (1 + \alpha_2 r_s) \ln\left(1 + \frac{1}{\xi}\right),$$

$$r_s = \left(\frac{3}{4\pi n_{\mathrm{e}} a_0^3}\right)^{\frac{1}{3}}, \tag{S10}$$

$$\xi = 2\alpha_1 \left(\alpha_3 r_s^{\frac{1}{2}} + \alpha_4 r_s + \alpha_5 r_s^{\frac{3}{2}} + \alpha_6 r_s^2\right),$$

with $\alpha_1 = 0.0310907, \alpha_2 = 0.21370, \alpha_3 = 7.5957, \alpha_4 = 3.5876, \alpha_5 = 1.6382, \alpha_6 = 0.49294$. θ_C is a gradient coefficient in the correlation energy.

$F_\otimes$ describes specific interactions between solution particles and the electron gas, which should be functionals of the electron density and the density of solution particles. Fundamentally, $F_\otimes$ needs to be determined consistently from the electronic functionals in $F_\mathbb{Q}$. Here, we use the following empirical relation,

$$F_\otimes = \sum_{l=1}^{N_\mathbb{C}} \int d^3r\, n_l w_l, \tag{S11}$$

where w_l characterizes short-ranged interactions between the metal and solution particles, for which we use a Morse potential,

$$w_l(\vec{r}) = D_l(\exp(-2\beta_l d(\vec{r})) - 2\exp(-\beta_l d(\vec{r}))), \tag{S12}$$

with D_l being the well depth, β_l a coefficient controlling the well width, $d(\vec{r})$ the distance from $\vec{r}$ to the metal surface. When $\vec{r}$ is within the metal, $d(\vec{r})$ is negative and $w_l(\vec{r})$ becomes very positive, meaning that solution particles have a negligible probability there.

Combined, the volumetric grand potential g of the EDL is written as,

$$\begin{aligned} g = {} & e_{au} a_0^{-3}\left(t_{TF}(1 + \theta_T s^2) + u_X^0(1 + \theta_X s^2) + u_C^0 + \theta_C n_e a_0^3 t^2\right) \\ & + (n_{cc} - n_e) e_0 \phi - \frac{1}{2}\epsilon_{op}(\nabla\phi)^2 \end{aligned} \tag{S13}$$

$$+\sum_{l=1}^{N_{\mathbb{C}}} n_l\left(w_l+\delta(l\in M)q_l\phi-\delta(l\in S)\beta^{-1}\ln\frac{\sinh(\beta p_l|\nabla\phi|)}{\beta p_l|\nabla\phi|}\right)$$

$$+\sum_{l=1}^{N_{\mathbb{C}}}\beta^{-1}n_l\left(\ln(n_l\Lambda_l^3)-1\right)+\Phi_{\text{ex}}(\{n_l\})-n_{\text{e}}\tilde{\mu}_{\text{e}}-\sum_{l=1}^{N_{\mathbb{C}}}n_l\tilde{\mu}_l,$$

which is an orbital-free, hybrid density-potential functional.

Variational analysis

Variational analysis of g in terms of ϕ gives,

$$\frac{\partial g}{\partial\phi}-\nabla\left(\frac{\partial g}{\partial\nabla\phi}\right)=0, \tag{S14}$$

leading to,

$$-\nabla[\epsilon_{\text{eff}}\nabla\phi]=e_0(n_{\text{cc}}-n_{\text{e}})+\sum_{l=1}^{N_{\mathbb{C}}}\delta(l\in \text{M})n_lq_l, \tag{S15}$$

which is the Poisson equation with an effective dielectric constant as in ref. [9-10]

$$\epsilon_{\text{eff}}=\epsilon_{\text{op}}+\sum_{l=1}^{N_{\mathbb{C}}}\frac{\delta(l\in\text{S})n_lp_l}{|\nabla\phi|}\left[\coth(\beta p_l|\nabla\phi|)-\frac{1}{\beta p_l|\nabla\phi|}\right]. \tag{S16}$$

Variational analysis of g in terms of particle number densities n_l should be divided into two cases. For the case of electrons, we obtain,

$$\nabla\left[\frac{\partial(t_{\mathrm{ni}}+u_{\mathrm{X}}+u_{\mathrm{C}})}{\partial\nabla n_{\mathrm{e}}}\right]=\frac{\partial(t_{\mathrm{ni}}+u_{\mathrm{X}}+u_{\mathrm{C}})}{\partial n_{\mathrm{e}}}-e_0\phi-\tilde{\mu}_{\mathrm{e}}. \tag{S17}$$

The electrochemical potential of electrons can be tuned by the electrode potential, ϕ_{M},

$$\tilde{\mu}_{\mathrm{e}}=\mu_{\mathrm{e}}-e_0\phi_{\mathrm{M}}, \tag{S18}$$

with μ_{e} the chemical potential of electrons.

Eqs.(S15) and (S17) constitute the basic set of differential equations controlling the EDL. Next, we need to derive expressions of n_l as functions of ϕ. Variational analysis of g in terms of charged particles in solution gives,

$$\begin{aligned} &w_l+\delta(l\in\mathrm{M})q_l\phi-\delta(l\in\mathrm{S})\beta^{-1}\ln\frac{\sinh\left(\beta p_l|\nabla\phi|\right)}{\beta p_l|\nabla\phi|} \\ &+\beta^{-1}\ln\left(n_l\Lambda_l^3\right)+\mu_l^{\mathrm{ex}}-\tilde{\mu}_l=0, \end{aligned} \tag{S19}$$

where μ_l^{ex} is the excess chemical potential, given by,

$$\mu_l^{\mathrm{ex}}=\frac{\delta\Phi_{\mathrm{ex}}}{\delta n_l}. \tag{S20}$$

In this work, the excess term Φ_{ex} is described at the level of Bikerman theory. Bikerman developed a lattice gas approach to calculate the mixing entropy of the electrolyte solution.[11] The Bikerman theory assumes that all charged particles have the same size Λ_{B}.The maximum number density is $n_{\max}=(\Lambda_{\mathrm{B}})^{-3}$. The Bikerman theory gives,

$$\mu_l^{\mathrm{ex}} = \beta^{-1}\ln\left(\frac{1}{1-\sum_{l=1}^{N_{\mathbb{C}}} n_l \Lambda_{\mathrm{B}}^3}\right) \quad \text{(S21)}$$

A more advanced description is the fundamental measure theory (FMT),[12-14], which has been compared with the Bikerman theory in a previous work.[1]

From Eq.(S19), n_l is given by,

$$\frac{n_l \Lambda_l^3}{1-\sum_{l=1}^{N_{\mathbb{C}}} n_l \Lambda_{\mathrm{B}}^3} = \Theta_l \exp\left(\beta\tilde{\mu}_l\right), \quad \text{(S22)}$$

where thermodynamic factors are given by

$$\Theta_l = \exp\left(-\beta\left(\delta(l\in \mathrm{M}) q_l\phi - \delta(l\in \mathrm{S})\beta^{-1}\ln\frac{\sinh\left(\beta p_l|\nabla\phi|\right)}{\beta p_l|\nabla\phi|} + w_l\right)\right). \quad \text{(S23)}$$

Equation (S25) shall be valid also in the solution bulk where $\Theta_l = 1$, and $\tilde{\mu}_l$ is uniform in the electrolyte solution. Combining these two conditions, we have the following equality,

$$\frac{n_l \Lambda_l^3}{1-\sum_{l=1}^{N_{\mathbb{C}}} n_l \Lambda_{\mathrm{B}}^3} = \Theta_l \frac{n_l^b \Lambda_l^3}{1-\sum_{l=1}^{N_{\mathbb{C}}} n_l^b \Lambda_{\mathrm{B}}^3}. \quad \text{(S24)}$$

We have,

$$n_l = n_{\max}\frac{\chi_l \Theta_l}{1+\sum_{l=1}^{N_{\mathbb{C}}} \chi_l(\Theta_l - 1)}. \quad \text{(S25)}$$

with dimensionless bulk number densities $\chi_l = n_l^b/n_{\max}$.

Equation (S25) can be extended, in a phenomenological manner, to scenarios of unequal sizes,

$$n_l = n_{\max}\frac{\chi_l\Theta_l}{\Omega}. \tag{S26}$$

where $\Omega = 1 + \sum_{l=1}^{N_{\mathbb{C}}}\gamma_l\chi_l(\Theta_l - 1)$ is the normalization factor, and γ_l is the relative size of particles of the type l referenced to Λ_B.

Formal transformation

To facilitate numerical implementation, we manipulate the controlling equations in Eq.(S15) and (S17) further. We can rewrite Eq. (S17) in terms of the dimensionless electron density, $\bar{n}_e = n_e a_0^3$,

$$\bar{\nabla}\left[\frac{\partial(t_{\mathrm{ni}} + u_{\mathrm{X}} + u_{\mathrm{C}})}{\partial\bar{\nabla}\bar{n}_e}\right] = \frac{\partial(t_{\mathrm{ni}} + u_{\mathrm{X}} + u_{\mathrm{C}})}{\partial\bar{n}_e} - a_0^{-3}(e_0\phi + \tilde{\mu}_{\mathrm{e}}) \tag{S27}$$

where the terms are obtained as,

$$\frac{\partial(t_{\mathrm{ni}} + u_{\mathrm{X}} + u_{\mathrm{C}})}{\partial\bar{\nabla}\bar{n}_e} = \frac{\partial(t_{\mathrm{ni}} + u_{\mathrm{X}} + u_{\mathrm{C}})}{\partial s^2}\frac{\partial s^2}{\partial\bar{\nabla}\bar{n}_e}$$

$$= \frac{e_{\mathrm{au}}a_0^{-3}(\theta_{\mathrm{T}}t_{\mathrm{TF}} + \theta_{\mathrm{XC}}u_{\mathrm{X}}^0)}{2(3\pi^2)^{\frac{2}{3}}(\bar{n}_e)^{\frac{8}{3}}}\bar{\nabla}\bar{n}_e, \tag{S28}$$

with $\theta_{\mathrm{XC}} = \theta_{\mathrm{X}} - \frac{\pi^2}{3}\theta_{\mathrm{C}}$, and

$$\frac{\partial(t_{\mathrm{ni}} + u_{\mathrm{X}} + u_{\mathrm{C}})}{\partial\bar{n}_e} \tag{S29}$$

$$= e_{\mathrm{au}} a_0^{-3} [(1 + \theta_{\mathrm{T}} s^2) \frac{\partial t_{\mathrm{TF}}}{\partial \bar{n}_e} + (1 + \theta_{\mathrm{XC}} s^2) \frac{\partial u_{\mathrm{X}}^0}{\partial \bar{n}_e} + \frac{\partial u_{\mathrm{C}}^0}{\partial \bar{n}_e} + (\theta_{\mathrm{T}} t_{\mathrm{TF}} + \theta_{\mathrm{X}}' u_{\mathrm{X}}^0) \frac{\partial s^2}{\partial \bar{n}_e}],$$

with,

$$\frac{\partial t_{\mathrm{TF}}}{\partial \bar{n}_e} = \frac{1}{2} (3\pi^2)^{\frac{2}{3}} (\bar{n}_e)^{\frac{2}{3}} \tag{S30}$$

$$\frac{\partial s^2}{\partial \bar{n}_e} = -\frac{8}{3} \frac{(\bar{\nabla} \bar{n}_e)^2}{4(3\pi^2)^{\frac{2}{3}} (\bar{n}_e)^{\frac{11}{3}}} = \frac{-8}{3\bar{n}_e} s^2 \tag{S31}$$

$$\frac{\partial u_{\mathrm{X}}^0}{\partial \bar{n}_e} = -\left(\frac{3}{\pi}\right)^{\frac{1}{3}} (\bar{n}_e)^{\frac{1}{3}} \tag{S32}$$

$$\frac{\partial u_{\mathrm{C}}^0}{\partial \bar{n}_e} = -2\alpha_1 (1 + \alpha_2 r_s) \ln\left(1 + \frac{1}{\xi}\right) - 2\alpha_1 \bar{n}_e \left(-\frac{1}{3}\left(\frac{3}{4\pi}\right)^{\frac{1}{3}} (\bar{n}_e)^{-\frac{4}{3}}\right) \cdot$$

$$\left(\alpha_2 \ln\left(1 + \frac{1}{\xi}\right) - \frac{(1 + \alpha_2 r_s)}{\xi(1 + \xi)} \alpha_1 \left(\alpha_3 r_s^{-\frac{1}{2}} + 2\alpha_4 + 3\alpha_5 r_s^{\frac{1}{2}} + 4\alpha_6 r_s\right)\right)$$

(S33)

$$= -2\alpha_1 (1 + \alpha_2 r_s) \ln\left(1 + \frac{1}{\xi}\right)$$

$$+ \frac{2\alpha_1 r_s}{3} \left(\alpha_2 \ln\left(1 + \frac{1}{\xi}\right) - \frac{\alpha_1 (1 + \alpha_2 r_s)}{\xi(1 + \xi)} \left(\alpha_3 r_s^{-\frac{1}{2}} + 2\alpha_4 + 3\alpha_5 r_s^{\frac{1}{2}} + 4\alpha_6 r_s\right)\right),$$

We expand the term on the right most side of Eq.(S28),

$$\overline{\nabla}\left[\frac{\left(\theta_{\mathrm{T}}t_{\mathrm{TF}}+\theta'_{\mathrm{X}}u_{\mathrm{X}}^{0}\right)}{(\overline{n}_e)^{\frac{8}{3}}}\overline{\nabla}\overline{n}_e\right]=\frac{\left(\theta_{\mathrm{T}}t_{\mathrm{TF}}+\theta_{\mathrm{XC}}u_{\mathrm{X}}^{0}\right)}{(\overline{n}_e)^{\frac{8}{3}}}\overline{\nabla}\overline{\nabla}\overline{n}_e-\overline{\nabla}\left[\frac{\left(\theta_{\mathrm{T}}t_{\mathrm{TF}}+\theta_{\mathrm{XC}}u_{\mathrm{X}}^{0}\right)}{(\overline{n}_e)^{\frac{8}{3}}}\right]\overline{\nabla}\overline{n}_e$$

$$=\frac{\left(\theta_{\mathrm{T}}t_{\mathrm{TF}}+\theta_{\mathrm{XC}}u_{\mathrm{X}}^{0}\right)}{(\overline{n}_e)^{\frac{8}{3}}}\overline{\nabla}\overline{\nabla}\overline{n}_e-(\overline{n}_e)^{-\frac{8}{3}}\left(\theta_{\mathrm{T}}\frac{\partial t_{\mathrm{TF}}}{\partial\overline{n}_e}+\theta_{\mathrm{XC}}\frac{\partial u_{\mathrm{X}}^{0}}{\partial\overline{n}_e}\right)(\overline{\nabla}\overline{n}_e)^2 \tag{S34}$$

$$-\frac{8}{3}(\overline{n}_e)^{-\frac{11}{3}}\left(\theta_{\mathrm{T}}t_{\mathrm{TF}}+\theta_{\mathrm{XC}}u_{\mathrm{X}}^{0}\right)(\overline{\nabla}\overline{n}_e)^2$$

Combining Eq.(S27),(S28),(S29), we get

$$\overline{\nabla}\overline{\nabla}\overline{n}_e=\frac{2\left(3\pi^2\right)^{\frac{2}{3}}(\overline{n}_e)^{\frac{8}{3}}}{\theta_{\mathrm{T}}t_{\mathrm{TF}}+\theta'_{\mathrm{X}}u_{\mathrm{X}}^{0}}[\left(1+\theta_{\mathrm{T}}s^2\right)\frac{\partial t_{\mathrm{TF}}}{\partial\overline{n}_e}+\left(1+\theta_{\mathrm{XC}}s^2\right)\frac{\partial u_{\mathrm{X}}^{0}}{\partial\overline{n}_e}+\frac{\partial u_{\mathrm{C}}^{0}}{\partial\overline{n}_e}$$

$$+\left(\theta_{\mathrm{T}}t_{\mathrm{TF}}+\theta_{\mathrm{XC}}u_{\mathrm{X}}^{0}\right)\frac{\partial s^2}{\partial\overline{n}_e}-\frac{(e_0\phi+\tilde{\mu}_{\mathrm{e}})}{e_{\mathrm{au}}}] \tag{S35}$$

$$+\frac{8}{3}(\overline{n}_e)^{-1}(\overline{\nabla}\overline{n}_e)^2-\frac{\theta_{\mathrm{T}}\frac{\partial t_{\mathrm{TF}}}{\partial\overline{n}_e}+\theta_{\mathrm{XC}}\frac{\partial u_{\mathrm{X}}^{0}}{\partial\overline{n}_e}}{\theta_{\mathrm{T}}t_{\mathrm{TF}}+\theta_{\mathrm{XC}}u_{\mathrm{X}}^{0}}(\overline{\nabla}\overline{n}_e)^2$$

Substituting Eq.(S31) into Eq.(S35), and using $s=|\overline{\nabla}\overline{n}_e|\Big/\left(2\left(3\pi^2\right)^{\frac{1}{3}}(\overline{n}_e)^{\frac{4}{3}}\right)$, we get,

$$\overline{\nabla}\overline{\nabla}\overline{n}_e=\frac{2\left(3\pi^2\right)^{\frac{2}{3}}(\overline{n}_e)^{\frac{8}{3}}}{\theta_{\mathrm{T}}t_{\mathrm{TF}}+\theta_{\mathrm{XC}}u_{\mathrm{X}}^{0}}\left(\frac{\partial t_{\mathrm{TF}}}{\partial\overline{n}_e}+\frac{\partial u_{\mathrm{X}}^{0}}{\partial\overline{n}_e}+\frac{\partial u_{\mathrm{C}}^{0}}{\partial\overline{n}_e}-\frac{(e_0\phi+\tilde{\mu}_{\mathrm{e}})}{e_{\mathrm{au}}}\right) \tag{S36}$$

$$+\left(\frac{4}{3}(\bar{n}_e)^{-1}-\frac{\theta_{\mathrm{T}}\frac{\partial t_{\mathrm{TF}}}{\partial \bar{n}_e}+\theta_{\mathrm{XC}}\frac{\partial u_{\mathrm{X}}^{0}}{\partial \bar{n}_e}}{2\left(\theta_{\mathrm{T}}t_{\mathrm{TF}}+\theta_{\mathrm{XC}}u_{\mathrm{X}}^{0}\right)}\right)(\bar{\nabla}\bar{n}_e)^2$$

Since, $\frac{\partial t_{\mathrm{TF}}}{\partial \bar{n}_e}=\frac{5t_{\mathrm{TF}}}{3\bar{n}_e},\frac{\partial u_{\mathrm{X}}^{0}}{\partial \bar{n}_e}=\frac{4u_{\mathrm{X}}^{0}}{3\bar{n}_e}$, we obtain

$$\frac{4}{3}(\bar{n}_e)^{-1}-\frac{\theta_{\mathrm{T}}\frac{\partial t_{\mathrm{TF}}}{\partial \bar{n}_e}+\theta_{\mathrm{XC}}\frac{\partial u_{\mathrm{X}}^{0}}{\partial \bar{n}_e}}{2\left(\theta_{\mathrm{T}}t_{\mathrm{TF}}+\theta_{\mathrm{XC}}u_{\mathrm{X}}^{0}\right)}=\frac{\left(\theta_{\mathrm{T}}t_{\mathrm{TF}}+\frac{4}{3}\theta_{\mathrm{XC}}u_{\mathrm{X}}^{0}\right)}{2\bar{n}_e\left(\theta_{\mathrm{T}}t_{\mathrm{TF}}+\theta_{\mathrm{XC}}u_{\mathrm{X}}^{0}\right)} \tag{S37}$$

In the end of the day, we reformulate the controlling equation for the electron density as,

$$\bar{\nabla}\bar{\nabla}\bar{n}_e=\frac{20}{3}\bar{n}_e\frac{\omega}{\theta_{\mathrm{T}}\omega-\theta_{\mathrm{XC}}}\left(\frac{\partial t_{\mathrm{TF}}}{\partial \bar{n}_e}+\frac{\partial u_{\mathrm{X}}^{0}}{\partial \bar{n}_e}+\frac{\partial u_{\mathrm{C}}^{0}}{\partial \bar{n}_e}-\frac{(e_0\phi+\tilde{\mu}_{\mathrm{e}})}{e_{\mathrm{au}}}\right)$$
$$+\frac{\left(\theta_{\mathrm{T}}\omega-\frac{4}{3}\theta_{\mathrm{XC}}\right)}{2\bar{n}_e(\theta_{\mathrm{T}}\omega-\theta_{\mathrm{XC}})}(\bar{\nabla}\bar{n}_e)^2 \tag{S38}$$

with $\omega=\frac{2}{5}\pi^{\frac{5}{3}}3^{\frac{1}{3}}(\bar{n}_e)^{\frac{1}{3}}$

Eq.(S38) was solved in Matlab in previous works.[1, 15-16] It was found that special treatments are needed for a stable and convergent numerical solution. Specifically, zero or negative $\bar{n}_e$ must be avoided as $\bar{n}_e$ occurs in the denominator and fractional exponents of $\bar{n}_e$ are required in many places. A positive-defined $\bar{n}_e$, $\bar{n}_e^{\mathrm{mod}}$ $=|\bar{n}_e|+\bar{n}_e^{\mathrm{lb}}$ with $\bar{n}_e^{\mathrm{lb}}$ being a positive lower bound was used for this end.

In this work, we propose a new numerical scheme in which we solve for $\psi = (\overline{n}_e)^{\frac{1}{3}}$ rather than $\overline{n}_e$. This way, we can get rid of issues related to fractional exponents of $\overline{n}_e$. With $\overline{\nabla}\overline{n}_e = \overline{\nabla}\psi^3 = 3\psi^2\overline{\nabla}\psi$, and $\overline{\nabla}\overline{\nabla}\overline{n}_e = \overline{\nabla}(3\psi^2\overline{\nabla}\psi) = 3\psi^2\overline{\nabla}\overline{\nabla}\psi + 6\psi(\overline{\nabla}\psi)^2$, we can transform Eq. (S38) to,

$$\overline{\nabla}\overline{\nabla}\psi + \frac{\pi^{\frac{5}{3}}3^{\frac{1}{3}}\theta_{\mathrm{T}}}{5(\theta_{\mathrm{T}}\omega - \theta_{\mathrm{XC}})}(\overline{\nabla}\psi)^2 = \frac{20}{9}\psi\frac{\omega}{\theta_{\mathrm{T}}\omega - \theta_{\mathrm{XC}}}\left(\frac{\partial t_{\mathrm{TF}}}{\partial \overline{n}_e} + \frac{\partial u_{\mathrm{X}}^0}{\partial \overline{n}_e} + \frac{\partial u_{\mathrm{C}}^0}{\partial \overline{n}_e} - \frac{(e_0\phi + \tilde{\mu}_{\mathrm{e}})}{e_{\mathrm{au}}}\right) \tag{S39}$$

Next, we turn to the controlling equation for the electric potential. We define dimensionless variables, marked with an over-bar, as follows,

$$\overline{n}_l = n_l(a_0)^3, \overline{x} = \frac{x}{a_0}, \overline{\phi} = \frac{e_0\phi}{k_B T}, \overline{p} = \frac{p}{e_0 a}, \overline{q}_l = \frac{q_l}{e_0}, \overline{\epsilon}_{\mathrm{op}} = \frac{\epsilon_{\mathrm{op}}}{\epsilon_0}$$

with ϵ_0 being the dielectric permittivity of vacuum.

We rewrite Eq.(S15) in a nondimensional form,

$$-\overline{\nabla}\left(\overline{\epsilon}_{\mathrm{op}}\overline{\nabla}\overline{\phi} + \sum_{l=1}^{N_{\mathbb{C}}}\delta(l \in \mathrm{S})\overline{n}_l\overline{p}_l\kappa\mathcal{L}_l\right) = \kappa\left((\overline{n}_{\mathrm{cc}} - \overline{n}_{\mathrm{e}}) + \sum_{l=1}^{N_{\mathbb{C}}}\delta(l \in \mathrm{M})\overline{n}_l\overline{q}_l\right), \tag{S40}$$

where $\kappa = \frac{e_0^2}{k_B T\epsilon_0 a_0}$ is a number derived from fundamental constants, and $\mathcal{L}_l = \coth\left(\overline{p_l E}\right) - \left(\overline{p_l E}\right)^{-1}$ is the Langevin function.

More details about numerical implementation

Initial guess

Numerical results of the model depend crucially on the initial guess. In this work, we use the following initial guess for the electron density and its gradient

$$\overline{n}_e(\overline{x}) = \frac{\overline{n}_{\mathrm{cc}}^0}{2}\mathrm{erfc}\big(\beta(\overline{x} - \overline{x}_{\mathrm{m}})\big) \tag{S41}$$

$$\overline{\nabla}\overline{n}_e(\overline{x}) = \frac{\beta\overline{n}_{\mathrm{cc}}^0}{\sqrt{\pi}}\exp\big(-\beta^2(\overline{x} - \overline{x}_{\mathrm{m}})^2\big) \tag{S42}$$

Correspondingly, the initial guess for the electric potential is obtained from solving a simplified version of Eq.(S40), $-\overline{\nabla}^2\overline{\phi} = \kappa(\overline{n}_{\mathrm{cc}} - \overline{n}_{\mathrm{e}})$,

$$\overline{\phi}(\overline{x}) = \frac{\kappa\overline{n}_{\mathrm{cc}}^0}{8\beta^2}\begin{pmatrix} -4\beta^2(\overline{x} - \overline{x}_{\mathrm{M}})^2\theta(\overline{x}_{\mathrm{M}} - \overline{x}) + \big(1 + 2\beta^2(\overline{x} - \overline{x}_{\mathrm{M}})^2\big)\mathrm{erfc}\big(\beta(\overline{x} - \overline{x}_{\mathrm{M}})\big) \\ -2\frac{\beta(\overline{x} - \overline{x}_{\mathrm{M}})}{\sqrt{\pi}}\exp\big(-\beta^2(\overline{x} - \overline{x}_{\mathrm{M}})^2\big) \end{pmatrix} \tag{S43}$$

and the corresponding potential gradient reads,

$$\overline{\nabla}\overline{\phi}(\overline{x}) = -\frac{\kappa\overline{n}_{\mathrm{cc}}^0}{2\beta}\begin{pmatrix} 2\beta(\overline{x} - \overline{x}_{\mathrm{M}})\theta(\overline{x}_{\mathrm{M}} - \overline{x}) + \frac{1}{\sqrt{\pi}}\exp\big(-\beta^2(\overline{x} - \overline{x}_{\mathrm{M}})^2\big) \\ -\beta(\overline{x} - \overline{x}_{\mathrm{M}})\mathrm{erfc}\big(\beta(\overline{x} - \overline{x}_{\mathrm{M}})\big) \end{pmatrix} \tag{S44}$$

Tips for numerical stability and convergence

First, zero ψ must be avoided in Eq.(S10), where we use a positive-defined ψ, $\psi_{\mathrm{mod}} = \max(\psi, 10^{-5})$. Second, the exponential terms in Eq.(S23) could be infinitely large when the electric field and the electric potential have very large magnitudes. Therefore, we need to set an upper limit, say 10^{80}, for these terms to

prevent the occurrence of infinite numbers. Third, we use the asymptotic function of $\mathcal{L}_l = \coth\left(\overline{p_l}\overline{E}\right) - \left(\overline{p_l}\overline{E}\right)^{-1} \approx \frac{1}{3}\overline{p_l}\overline{E}$ when $\overline{p_l}\overline{E}$ is very small to avoid the occurrence of infinity when $\overline{E}$=0.

Supplementary figure

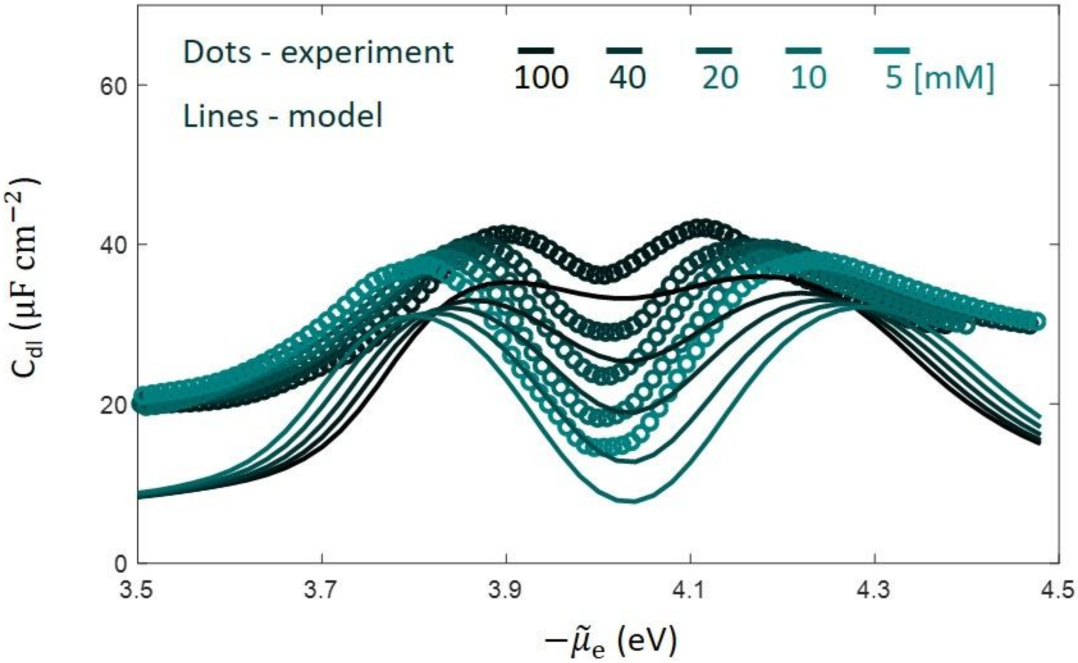

Fig. S 1Comparison between model and experimental results of the differential double layer capacitance (C_{dl}) of Ag(111)-KPF_6 aqueous interface at five concentrations indicated in the figure.

Building the model in COMSOL

Global parameters

Format: *Name-of-parameter Value-of-parameter Annotation*.

Save the following list in a text file under the name "Global parameters"

Do not change the blank between the name and the value.

```
a0 5.29e-11 [m], Bohr radius
a1 0.0310907 coefficients in correlation functional
a2 0.21370 coefficients in correlation functional
a3 7.5957 coefficients in correlation functional
a4 3.5876 coefficients in correlation functional
a5 1.6382 coefficients in correlation functional
a6 0.49294 coefficients in correlation functional
b 0.6 Exponential coefficient for initial guess, crucial parameter for convergence when theta_TF is big
betaa 1 coefficient in Morse potential of anions
betac 1 coefficient in Morse potential of cations
betaop 2 coefficient in empirical relation for optical permittivity
betas 1 coefficient in Morse potential of solvent
c_ion_b 100 [mM], ion concentration in solution bulk
chi_a n_ion_b/nmax fraction of anions
chi_c n_ion_b/nmax fraction of cations
chi_s n_s_b/nmax fraction of solvent dipoles
chi_v 0.05 Volume fraction of vacancies in bulk
da 4.0*1e-10/a0 dimensionless equilibrium potential of anions
Da Ds/6 Dimensionless interaction strength of metal-anion interaction
dc 4.0*1e-10/a0 dimensionless equilibrium potential of cations
Dc Ds/6 Dimensionless interaction strength of metal-cation interaction
ds 2.85*1e-10/a0 Metal-solvent distance
Ds 0.25*e0/kB/T Force constant of metal-solvent interaction
e0 1.6e-19 [C], electron charge
eau 27.2*e0 [J], energy constant from a.u. to SI
Ecr 1e-3 Dimensionless criterion used in Langvein function
```

Elb 1e-6 dimensionless lower bound of electric field in Langvein function
eps_op_M 1 optical dielectric constant of metal
eps_op_S 4 optical dielectric constant of solution
eps0 8.85e-12 [F/m], vacuum permittivity
gamma_a (2*ra/Rsol)^3 relative size of anions referenced to solvent
gamma_c (2*rc/Rsol)^3 relative size of cations referenced to solvent
gamma_s 1 relative size of solvent referenced to solvent
kappa e0^2/kB/T/eps0/a0 Constant
kB 1.38e-23 [J/K], Boltzmann constant
L_sol_w 30*ldebye/a0 dimensionless width of solution
L_sub_w 20 dimensionless width of substrate
ldebye sqrt(eps_op_S*eps0*kB*T/2/c_ion_b/NA/e0^2) [m], Debye length
n_ion_b c_ion_b*NA/nref dimensionless ion density in solution bulk
n_s_b 5.5e4*NA/nref dimensionless solvent density in solution bulk
NA 6.02e23 [1/mol], Avogadro number
ncc 0.408 dimensionless charge density of metal cationic cores
nmax (gamma_s*n_s_b+(gamma_c+gamma_a)*n_ion_b)/(1-chi_v) dimensionless lattice density in solution bulk
nref a0^-3 [1/m^3], reference density"
ps psd*3.33564e-30/e0/a0 dimensionless dipole moment of solvent
psd sqrt(3*(78.5-eps_op_S)*eps0*kB*T/(5.5e4*NA))/(3.33564e-30) [D], solvent dipole moment in Debye
ra 4 [A], radius of PF6- anions
rc 5 [A], radius of solvated K+ cations
Rsol 1e10*(1/5.5e4/NA)^(1/3) [A], diameter of water, ensuring bulk concentration of water
T 298 [K], Temperature
thetaC 0.046 gradient factor in correlation energy
thetaTF 2.08 gradient factor in kinetic energy
thetaX 0.1235 gradient factor in correlation energy
thetaXC thetaX-pi^2/3*thetaC effective gradient factor for XC
ucheme -4.05 [eV], emical potential of electrons"

Classical field

Save the following list in a text file under the name “Classical field”

```
dm x-L_sub_w closest distance to the metal cationic core
Ef min(abs(ux)+Elb,100) positive-ensured electric field
eps_op eps_op_M+(eps_op_S-eps_op_M)/2*(1-erf(-betaop*dm)) optical dielectric constant
epseff eps_op+kappa*ps^2*ns*mLg(abs(ux)*ps+Elb) dimensionless dielectric permittivity
na nmax*chi_a*TDa/TDt dimensionless number density of anions
nc nmax*chi_c*TDc/TDt dimensionless number density of cations
ns nmax*chi_s*TDs/TDt dimensionless number density of solvent dipoles
TDa min(exp(min(u,100)-w_srfa),1e80) min(u,100) - w_srf), 1e20)" "TD factor of anions"
TDc min(exp(min(-u,100)-w_srfc),1e80) thermodynamic factor of cations
TDs (sinh(ps*Ef)/(ps*Ef)*flc1hs(ps*Ef-Ecr,Elb)+(1+(ps*Ef)^2/6)*flc1hs(Ecr-ps*Ef,Elb))*exp(-w_srfs)
thermodynamic factor of solvent
TDt gamma_a*chi_a*TDa+gamma_c*chi_c*TDc+gamma_s*chi_s*TDs+chi_v sum of thermodynamic factors
w_srfa Srfa(dm) short-range force of metal-anion interactions
w_srfc Srfc(dm) short-range force of metal-cation interactions
w_srfs Srfs(dm) short-range force of metal-solvent interactions
```

Electronic part

Save the following list in a text file under the name “Electronic part”

```
ne v^3 electron density
rs (3/(4*pi))^(1/3)/vp effective radius
irs 2/5*pi^(5/3)*3^(1/3)*v irreversibe effective radius
xi 2*a1*(a3*(rs)^(1/2)+a4*rs+a5*(rs)^(3/2)+a6*(rs)^2) function of xi
gradTF 1/2*(3*pi^2)^(2/3)*v^2 chemical potential due to TF term
gradX -(3/pi)^(1/3)*v chemical potential due to X term
gradC -2*a1*(1+a2*rs)*log(1+1/xi)+2*a1*rs/3*(a2*log(1+1/xi)-a1*(1+a2*rs)/xi/(1+xi)*(a3*(rs)^(-
1/2)+2*a4+3*a5*(rs)^(1/2)+4*a6*rs)) chemical potential due to C term
mu_ueg gradTF+gradX+gradC-(u*kB*T+ucheme*e0)/eau electrochemical potential of electrons
c_new -1 diffusive coefficient for v
f_new v*20/9*irs*mu_ueg/(thetaTF*irs-thetaXC) source term in eq for v
beta thetaTF*2/5*pi^(5/3)*3^(1/3)/2/(thetaTF*irs-thetaXC)*vx convection coefficient for v
v_ll 1e-4 lower limit of v in vp
vp v*(v>v_ll)+v_ll positive ensured v
```

Initial guess

Save the following list in a text file under the name "Initial guess"

```
vinitial (ncc/2*(1-erf(b*dm)))^(1/3) initial guess of electron density
uinitial kappa*ncc/eps_op/8/b^2*(-4*b^2*dm^2*(dm<0)+(1+2*b^2*dm^2)*(1-erf(b*dm))-
2*b*dm/sqrt(pi)*exp(-b^2*dm^2)) initial guess of electric potential
```

A step-by-step tutorial of implementing the theory in COMSOL

New -> Blank Model -> Set the basics of the model -> save the model as “EDL_1D_Demo”

Note: the model is dimensionless and it’s important to set ‘None’ for the unit system.

Global Definitions -> Parameters -> Load from file -> Choose the file “Global parameters” that you have just created.

Right click “EDL_1D_Demo” -> Component -> 1D ->Component label: EDL

Definitions -> Variables -> label: Classical field -> load from file -> select the txt file “Classical field” that you have just created

Right click Definitions -> Variables -> label: Electronic part -> load from file -> select the txt file “Electronic part” that you have just created

Right click Definitions -> Variables -> label: Initial guess -> load from file -> select the txt file “Initial guess” that you have just created

Right click Definitions -> Function -> Analytic -> label: Modified Langvein function -> Function name: mLg -> Expression:

(coth(x)/x-1/(x^2))*flc1hs(x-Ecr, Elb)+1/3*flc1hs(Ecr-x,Elb)

Right click Definitions -> Function -> Analytic -> label: Short-range force between metal and solvent -> Function name: Srfs -> Arguments: d -> Expression:

Ds*(exp(-2*betas*(d-ds))-2*exp(-betas*(d-ds)))

Repeat creating metal-cation and metal-anion interactions and name the functions as Srfa and Srfc, respectively.

Now we define the geometry

Right click Geometry -> Interval -> Label: Metal part -> Right endpoint: L_sub_w

Right click Geometry -> Interval -> Label: Solution part -> Left endpoint: L_sub_w -> L_sub_w+L_sol_w

Build All Objects

Now we add physics

Right click EDL -> Add Physics -> Mathematics -> PDE Interfaces -> Coefficient Form PDE -> Add to Component

Click Coefficient Form PDE and modify the settings according to *Fig. S 2* and *Fig. S 3*, consecutively.

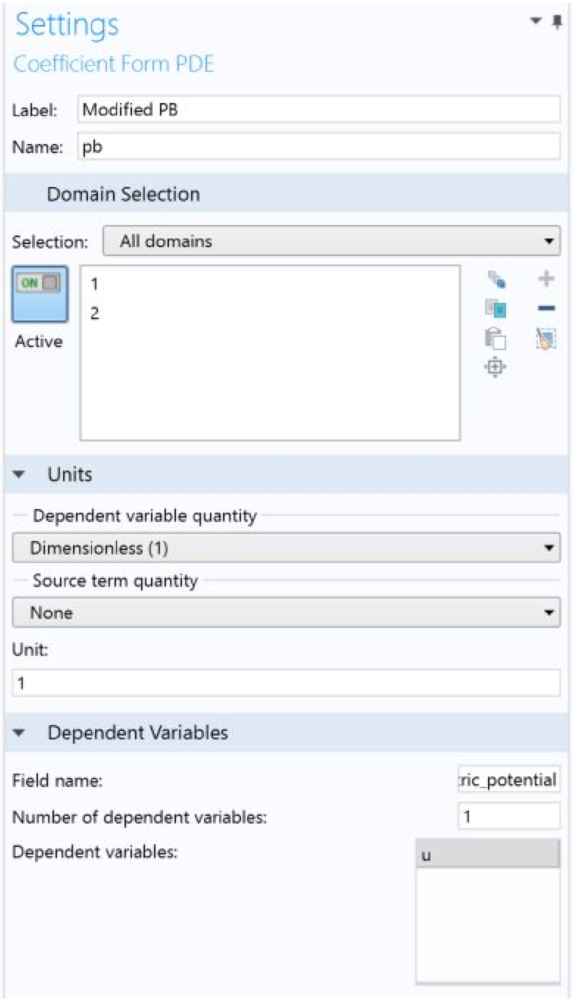

Fig. S 2 Modify settings of coefficient form PDE

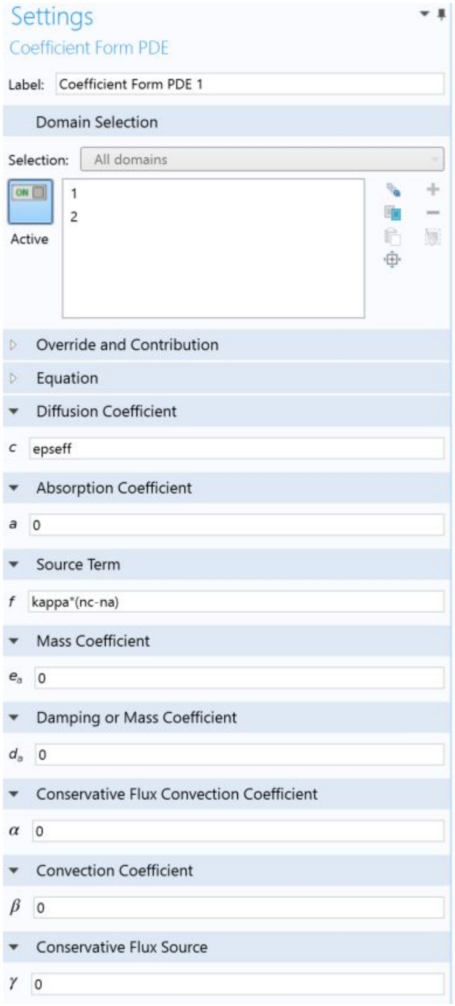

Fig. S 3 Modify coefficients of the PDE

Initial Values 1 -> initial value for u: uinitial

Right click Modified PB -> Source -> Label: electron charge -> Domain selection: All domains -> Source term: -kappa*v^3

Right click Modified PB -> Source -> Label: metal cationic charge -> Domain selection: 1 -> Source term: kappa*ncc

Right click Modified PB -> Dirichlet Boundary Condition -> Label: Potential reference -> Boundary selection: 3

Now the modified PB equation with all boundary conditions are well defined.

Next we do the same for the electron density equation.

Right click EDL -> Add Physics -> Mathematics -> PDE Interfaces -> Coefficient Form PDE -> Add to Component

Click Coefficient Form PDE and modify the settings according to Fig. S 4 and Fig. S 5, consecutively.

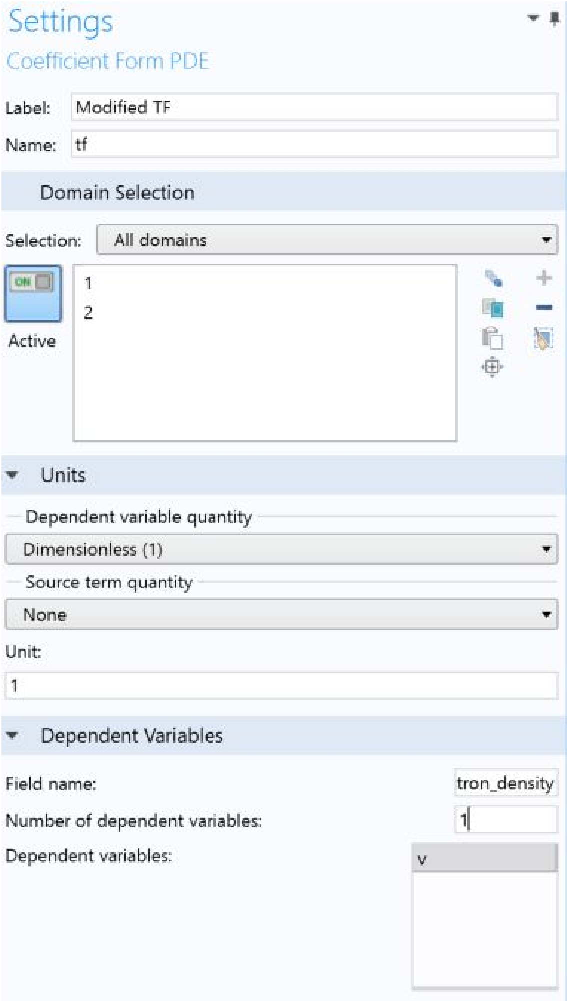

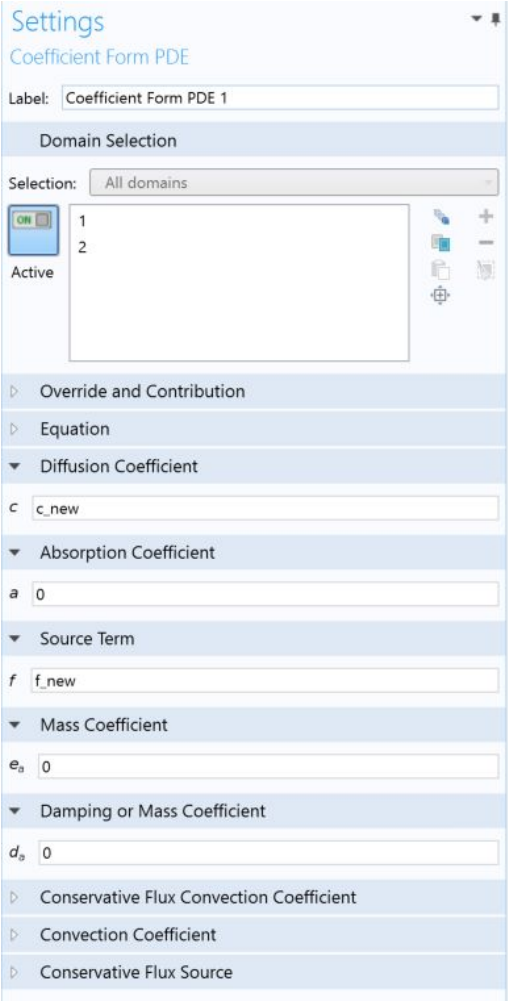

Fig. S 4 Modify settings of coefficient form PDE

Fig. S 5 Modify coefficients of the PDE

Initial Values 1 -> initial value for v: vinitial

Right click Modified TF -> Dirichlet Boundary Condition -> Label: solution bulk-> Boundary selection: 3

Right click Modified TF -> Dirichlet Boundary Condition -> Label: metal bulk-> Boundary selection: 1 -> Prescribed value of v: (ncc)^(1/3)

Now the modified TF equation with all boundary conditions are well defined.

Next, set Mesh as in *Fig. S 6*

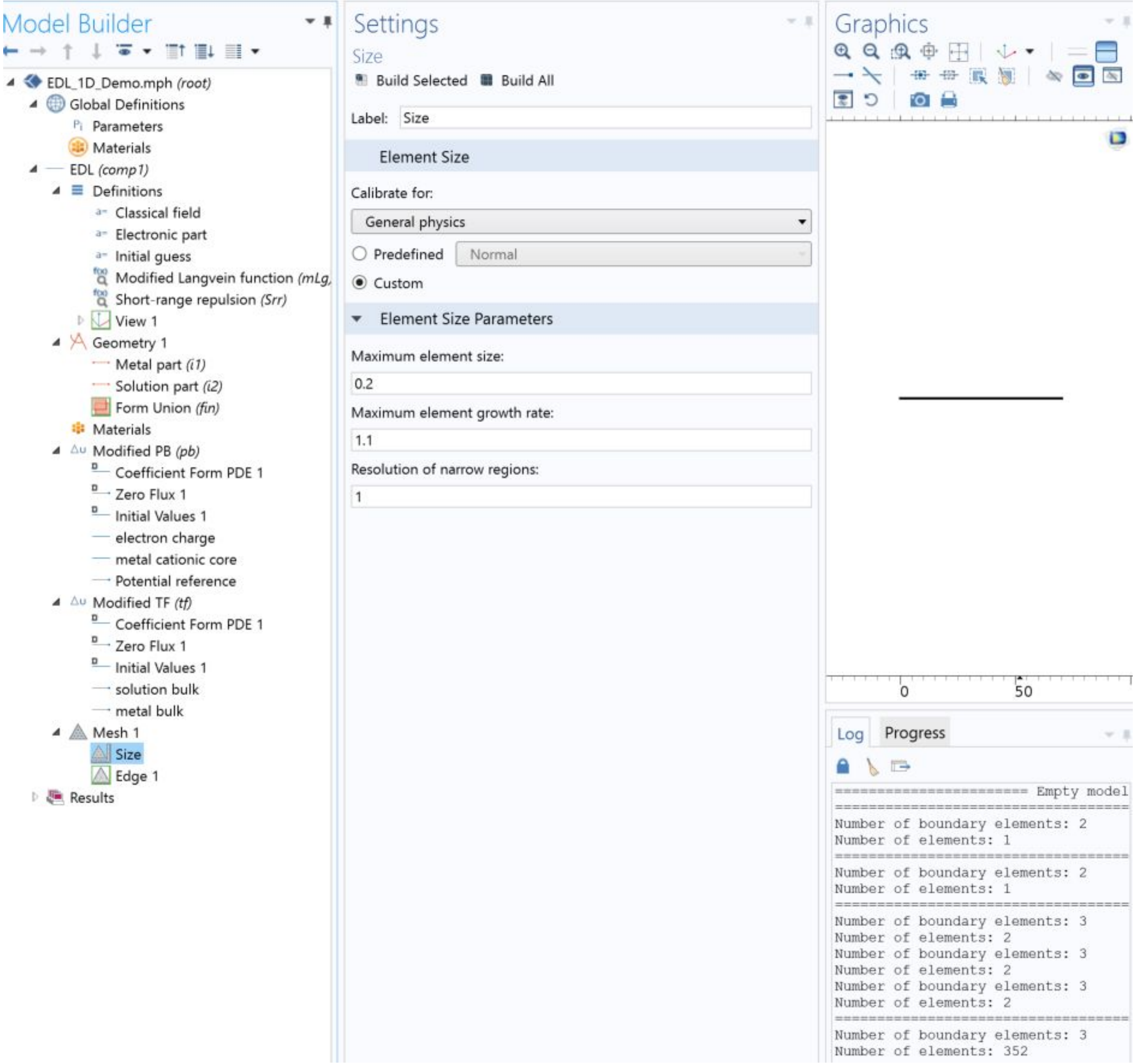

Fig. S 6 Model appearance after meshing

Now, we add a study

Right click EDL_1D_Demo -> Stationary

Right click Study 1 -> Parametric sweep -> add -> select ucheme -> Parameter value list: range(-3.5*e0,0.1*e0,-1.5*e0)

Right click Study 1 -> Show Default Solver -> Fully Coupled 1 -> modify settings as in

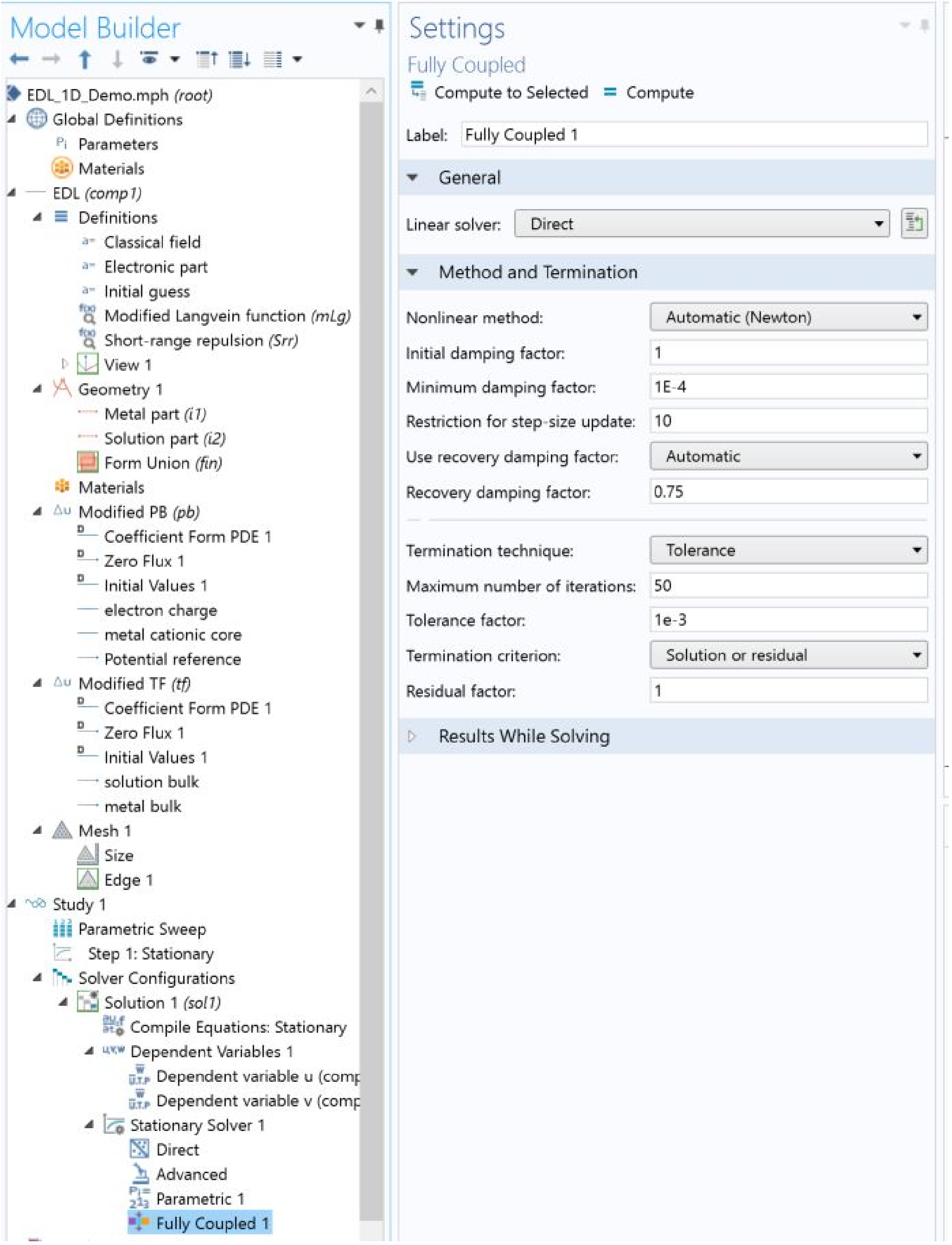

Fig. S 7 Setting the study

Now you can run your model.

Reference

1. Huang, J., Hybrid Density-Potential Functional Theory of Electric Double Layers. *Electrochimica Acta* **2021**, *389*, 138720.
2. Lue, L., A Variational Field Theory for Solutions of Charged, Rigid Particles. *Fluid Phase Equilibria* **2006**, *241*, 236-247.
3. Lundqvist, S.; March, N. H., *Theory of the Inhomogeneous Electron Gas*; Springer Science & Business Media, 2013.
4. Perdew, J. P.; Kurth, S., Density Functionals for Non-Relativistic Coulomb Systems in the New Century. In *A Primer in Density Functional Theory*, Fiolhais, C.; Nogueira, F.; Marques, M. A. L., Eds. Springer Berlin Heidelberg: Berlin, Heidelberg, 2003; pp 1-55.
5. Kohn, W.; Sham, L. J., Self-Consistent Equations Including Exchange and Correlation Effects. *Physical Review* **1965**, *140*, A1133-A1138.
6. Karasiev, V. V.; Calderín, L.; Trickey, S. B., Importance of Finite-Temperature Exchange Correlation for Warm Dense Matter Calculations. *Physical Review E* **2016**, *93*, 063207.
7. Thomas, L. H., The Calculation of Atomic Fields. *Math. Proc. Camb. Phil. Soc.* **1927**, *23*, 542-548.
8. Fermi, E., Eine Statistische Methode Zur Bestimmung Einiger Eigenschaften Des Atoms Und Ihre Anwendung Auf Die Theorie Des Periodischen Systems Der Elemente. *Z. Phys.* **1928**, *48*, 73-79.
9. Abrashkin, A.; Andelman, D.; Orland, H., Dipolar Poisson-Boltzmann Equation: Ions and Dipoles Close to Charge Interfaces. *Physical Review Letters* **2007**, *99*, 077801.
10. Nakayama, Y.; Andelman, D., Differential Capacitance of the Electric Double Layer: The Interplay between Ion Finite Size and Dielectric Decrement. *The Journal of Chemical Physics* **2015**, *142*, 044706.
11. Bikerman, J. J., Xxxix. Structure and Capacity of Electrical Double Layer. *Lond. Edinb. Dubl. Phil. Mag.* **1942**, *33*, 384-397.
12. Gillespie, D., A Review of Steric Interactions of Ions: Why Some Theories Succeed and Others Fail to Account for Ion Size. *Microfluidics and Nanofluidics* **2015**, *18*, 717-738.
13. Roth, R., Fundamental Measure Theory for Hard-Sphere Mixtures: A Review. *Journal of Physics: Condensed Matter* **2010**, *22*, 063102.
14. Rosenfeld, Y., Free-Energy Model for the Inhomogeneous Hard-Sphere Fluid Mixture and Density-Functional Theory of Freezing. *Physical Review Letters* **1989**, *63*, 980-983.
15. Huang, J.; Chen, S.; Eikerling, M., Grand-Canonical Model of Electrochemical Double Layers from a Hybrid Densitypotential Functional. *Journal of Chemical Theory and Computation* **2021**.
16. Huang, J.; Li, P.; Chen, S., Potential of Zero Charge and Surface Charging Relation of Metal-Solution Interphases from a Constant-Potential Jellium-Poisson-Boltzmann Model. *Physical Review B* **2020**, *101*, 125422.
